# Stretchable cyclodextrin slide-ring copolymer self-generating hydrogels for high-performance flexible electronics

**DOI:** 10.1039/d6sc00045b

**Published:** 2026-03-25

**Authors:** Xu Pan, Bo Wang, Xiao-Yong Yu, Jin-Long Yue, Xu Zhang, Chen Zhang, Ying-Ming Zhang, Yong Chen, Yu Liu

**Affiliations:** a College of Chemistry, State Key Laboratory of Elemento-Organic Chemistry, Nankai University Tianjin 300071 P. R. China chenyong@nankai.edu.cn yuliu@nankai.edu.cn; b Shandong Binzhou Zhiyuan Biotechnology Co., Ltd Shandong 256500 P. R. China

## Abstract

Supramolecular flexible electronic devices have received much attention due to their wide applications in the fields of chemistry, physics, materials and biology. Herein, we reported a slide-ring supramolecular hydrogel prepared by a thermal-initiated polymerization of acrylamide (AM), acrylic acid (AA), poly(3,4-ethylenedioxythiophene):poly(styrenesulfonate) (PEDOT:PSS) and a low-coverage cyclodextrin polyrotaxane as a cross-linker, displaying not only good mechanical properties due to its flexible structure, but also a notable self-generating capability. It is very important to note that the introduction of the low-coverage cyclodextrin polyrotaxane structure into the traditional polymer hydrogel resulted in significantly improved tensile strain, up to 12.48 times its original length, surpassing those of hydrogels based on traditional polyrotaxanes. The hydrogel exhibited a conductivity of 0.65 S m^−1^, ensuring that it is suitable for high-performance flexible electronic devices. The slide-ring hydrogel exhibited excellent resistive and triboelectric sensing ability and was used to prepare dual-modal flexible wearable sensors, which accurately captured both large-amplitude and high-frequency joint motion signals (finger, wrist, elbow, and knee), enabling human–computer interaction.

## Abstract 2

A slide-ring hydrogel was synthesized from AM, AA, PEDOT:PSS, and a low-coverage cyclodextrin polyrotaxane crosslinker, whose tensile strain was greatly enhanced. The hydrogel enabled dual-modal flexible wearable sensors for joint movement.

## Graphical abstract

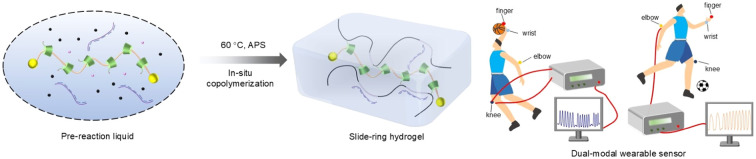

## Introduction

Flexible electronic devices have demonstrated broad application prospects in frontier fields such as wearable sensors,^1,2^ electronic skin^3,4^ and smart electronic textiles.^5,6^ The mechanical properties of flexible electronics can be significantly enhanced by introducing a supramolecular polyrotaxane structure, due to its exceptional flexibility,^7,8^ fatigue resistance^9,10^ and self-healing capability.^11,12^ In the polyrotaxane structure, cyclodextrin is one of the most important slide-ring molecules and can act as a ‘ring’ and slide along the polymer chain,^13–15^ thereby enhancing the stretchability and mechanical stability of the slide-ring hydrogel.^16–19^ On further introducing crosslinking sites onto the polyrotaxane, followed by polymerization with monomers, a supramolecular copolymer can be constructed. Recently, Zhao *et al.* prepared a flexible sensor by self-assembling acrylated β-cyclodextrin with bile acid *via* host–guest recognition, which was then photopolymerized with acrylamide.^20^ The hydrogel was capable of monitoring human electrocardiogram signals in real time, in which the polyrotaxane, a supramolecular slide-ring system with mechanically interlocked topology, exhibited excellent stretchability and fatigue resistance. Liu *et al.* reported a supramolecular hydrogel with an internal cross-linked network through coordination between calcium ions and carboxyl groups, and the stretchability and tensile toughness of the material were enhanced by incorporating the cyclodextrin slide-ring structure.^21^ The hydrogel exhibited a conductivity of 0.21 S m^−1^ and an open-circuit voltage of 420 V, demonstrating potential as a flexible electronic device for human–computer information transfer. Bai *et al.* fabricated a conductive hydrogel using 2-methoxyethyl acrylate, *N*-allylthiourea and a cyclodextrin polyrotaxane cross-linker.^22^ The synergistic effect of multiple hydrogen-bonding interactions within the hydrogel network ensured its stability under various environmental conditions, whose stability and functionality were retained at high (50 °C) and low (−42 °C) temperatures, enabling reliable motion sensing even underwater, particularly in seawater at 2 °C for winter swimming applications. Although traditional rotaxane structures have been widely adopted, their applications are customarily limited due to relatively low water solubility and modest improvement in mechanical properties.^23–26^ In contrast, low-coverage cyclodextrin polyrotaxanes, by reducing the number of threaded cyclodextrins, exhibit decreased entropic repulsion among cyclodextrins along the polymer backbone,^27,28^ which allows active sliding when subjected to high strain. Though low-coverage cyclodextrin polyrotaxanes can further enhance the flexibility of polymer materials, their application has been rarely reported to the best of our knowledge.

**Scheme 1:**
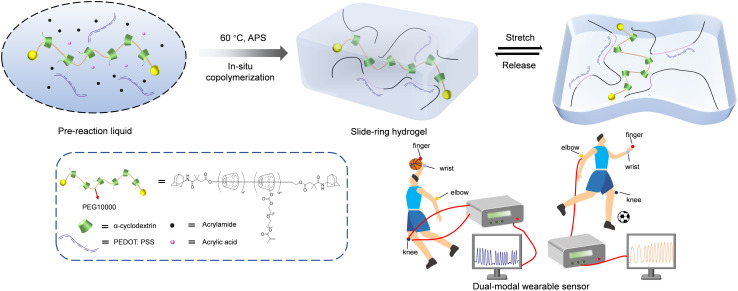
Construction of a stretchable slide-ring hydrogel (formation of a three-dimensional network structure by thermal polymerization).

Herein, we reported a low-coverage supramolecular slide-ring hydrogel prepared by thermal-initiated polymerization of an acrylamide-acrylic acid mixture with poly(3,4-ethylenedioxythiophene):poly(styrenesulfonate) (PEDOT:PSS) and a polyrotaxane cross-linker. In this system, the acrylamide (AM) and acrylic acid (AA) served as monomers, the low-coverage polyrotaxane acted as the cross-linker, ammonium persulfate (APS) served as the initiator and PEDOT:PSS acted as the conductive component. While PEDOT:PSS offers high conductivity and excellent electrochemical stability,^29,30^ it is inherently brittle with poor processability.^31–34^ To address these limitations, we integrated it into low-coverage polyrotaxane hydrogel networks to improve its flexibility. The low-coverage polyrotaxane cross-linker was synthesized by threading α-cyclodextrins (α-CDs) along a poly(ethylene glycol) (PEG) chain, followed by capping with adamantane groups and was subsequently functionalized to introduce cross-linking sites. Compared to traditional polymer hydrogels, the introduction of the low-coverage polyrotaxane structure significantly enhanced both the stretchability and tensile toughness of the hydrogel, outperforming hydrogels based on traditional polyrotaxanes, ensuring that it is suitable for flexible electronic devices. The slide-ring hydrogel, with excellent mechanical and conductive properties, successfully combined resistive strain and triboelectric sensing modes, demonstrating outstanding performance in human–computer information transfer by effectively monitoring motions from various body sites (finger, wrist, elbow, and knee) (Scheme 1).

## Results and discussion

**Scheme 2:**
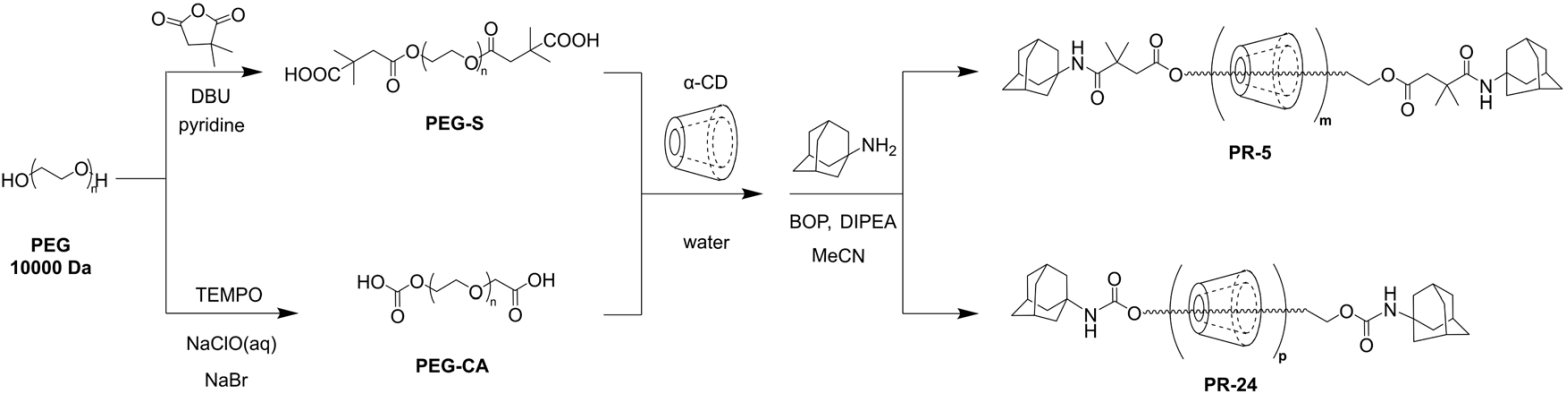
Synthetic schemes for two cyclodextrin polyrotaxanes with coverages of 5% and 24% (PR-5 and PR-24). Detailed procedures are shown in Schemes S1 and S2 in the SI.

The synthesis process of the low-coverage polyrotaxane crosslinker PR-5 was as follows (Schemes 2 and S1): PEG was modified with 2,2-dimethylsuccinic anhydride to prepare PEG-S (Fig. S1) and then α-CD was threaded through the PEG-S molecular chain, followed by end-capping with adamantane stoppers. According to the calculation based on peak areas of ^1^H NMR (Fig. S2), the PEG chain (*M*_w_ ≈ 10 000) contained 5.6 CD units. By comparing the 1 H protons of α-CD at 4.80 ppm (one α-CD unit possessed six 1 H protons) with the methylene protons of PEG at 3.50 ppm (one PEG chain possessed 909 methylene protons), the coverage ratio of the PEG chain was calculated to be 5% (based on one α-CD molecule occupying two polyethylene glycol units). In the 2D NOESY spectrum, signals assigned to 3-/5 H protons of α-CD at 3.75–3.5 ppm and PEG protons at 3.5 ppm exhibited clear NOE correlations, indicating that the PEG chain was included in the cavity of α-CD (Fig. S3). XPS spectra also displayed characteristic peaks of the polyrotaxane structure (Fig. S4). PEGMA-CDI was synthesized *via* the reaction between 1,1′-carbonyldiimidazole (CDI) and poly(ethylene glycol) methacrylate (PEGMA), and the formation was confirmed by ^1^H NMR and HRMS (Fig. S5 and S6). PEGMA-CDI randomly modified α-CD with PEGMA *via* substitution reactions between the imidazole groups and hydroxyls, forming the cross-linker molecule PR-PEGMA_*x*_ (*x* = 3, 8, 12), which enhanced water solubility and provided cross-linking functional sites, and the average number of PEGMA groups on polyrotaxane was calculated based on peak areas of ^1^H NMR (Fig. S7–S10). In the FT-IR spectra (Fig. S11), the disappearance of the peak at 3467 cm^−1^ corresponded to the substitution between CDI and PEGMA. The peaks at 1717 cm^−1^ and 1635 cm^−1^ were indicative of the successful grafting of methacrylate terminals, while the peak at 1745 cm^−1^ proved the formation of carbonate linkage, and differences in peak intensity further suggested the presence of various numbers of side chains in the cross-linker. To establish a control system, high-coverage polyrotaxane, PR-24, was synthesized (Schemes 2 and S2), and side chains were introduced to PR-24 using the same ratio as that of the aforementioned low-coverage polyrotaxane, to form the cross-linker PR-CA-PEGMA, which was verified by ^1^H NMR characterization (Fig. S12–S14).

A reaction system was established *via* the sequential addition of an aqueous dispersion of PEDOT:PSS and the cross-linker to an aqueous solution containing AM and AA monomers with a small quantity of the thermal initiator, APS, and the mixture was heated at 60 °C to facilitate polymerization, ultimately forming a blue-black transparent slide-ring hydrogel with a three-dimensional network structure. Both cross-links and hydrogen bonds endowed the hydrogel with a certain degree of self-healing ability. By comparing the dry weights of the hydrogel before and after immersion in excess water, the polymerization efficiency was calculated to be >95%, attributed to the good aqueous solubility of acrylamide, acrylic acid, and the polyrotaxane cross-linker. To further verify the influence of the polyrotaxane structure and its coverage on the hydrogel properties, two control systems were established: (1) using the aforementioned high-coverage polyrotaxane cross-linker and (2) using a conventional cross-linker poly(ethylene glycol) diacrylate (PEGDA) that lacked the polyrotaxane structure. Both control cross-linkers were used under reaction conditions identical to those of the low-coverage polyrotaxane cross-linking system to prepare corresponding control hydrogel samples, thereby excluding interference from factors other than cross-linker type on the hydrogel properties (Table S1).

**Fig. 1:**
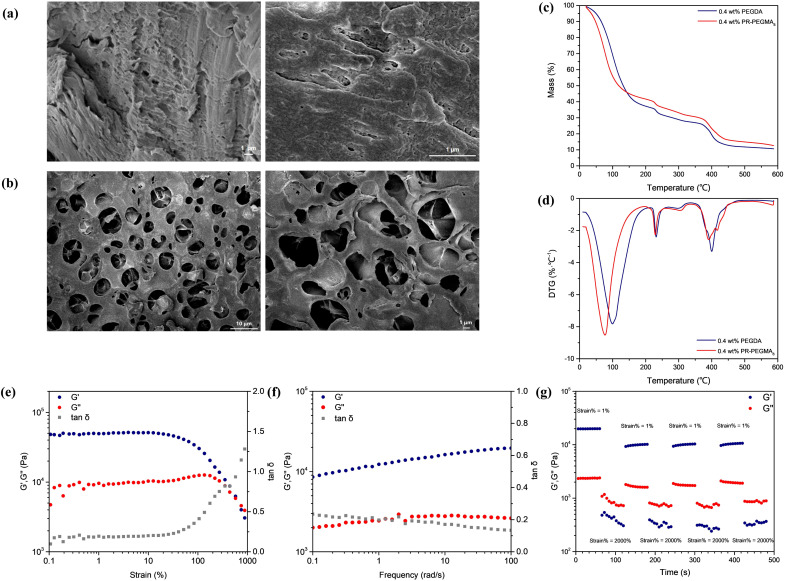
(a) Surface and (b) cross-sectional SEM image of a freeze-dried slide-ring hydrogel. (c) TGA and (d) DTG curve of the slide-ring hydrogel. (d) *G*′ (storage modulus), *G*″ (loss modulus) and tan *δ* (loss factor) as functions of strain. (e) *G*′, *G*″ and tan *δ* as functions of strain. (f) *G*′, *G*″ and tan *δ* as functions of frequency. (g) Rheological self-healing test with a fixed frequency of 1 Hz. Amplitude oscillatory strain levels of the hydrogel were turned between 1 and 2000% (PR-PEGMA_8_/PEDOT:PSS/AM/AA hydrogel, with 0.4 wt% PR-PEGMA_8_).

The cross-sectional SEM images of the freeze-dried slide-ring hydrogel showed a clear three-dimensional porous network structure, while the surface of the hydrogel was relatively flat (Fig. 1a and b). In the FT-IR spectrum of the freeze-dried hydrogel, the peak at 3325 cm^−1^ was assigned to the asymmetric stretching vibration of –NH_2_ and the symmetric stretching vibration of –NH–, the peak at 3189 cm^−1^ corresponded to the symmetric stretching vibration of –NH_2_, and the peaks at 2929 cm^−1^ and 1449 cm^−1^ were attributed to the stretching vibrations of CH_2_ and C–N. The strong peak at 1651 cm^−1^ was ascribed to the coupling of the C=O stretching vibrations from the carboxyl and amide functional groups, while the absence of a peak near 3100 cm^−1^ indicated the completion of the polymerization reaction (Fig. S15). In the Raman spectrum, the peak at 989 cm^−1^ was attributed to the oxyethylene ring deformation of PEDOT:PSS, while the peaks at 1367 cm^−1^ and 1566 cm^−1^ were associated with the asymmetric stretching deformations of the C_α_–C_α_ and C_β_–C_β_ bonds in the aromatic rings of PEDOT, and the peak at 1433 cm^−1^ was assigned to the symmetric stretching mode of C_α_=C_β_ (Fig. S16). XPS spectra further confirmed the presence of PEDOT:PSS, AM units, and AA units (Fig. S17). Thermogravimetric (TG) analysis of the hydrogel indicated three primary stages of mass loss: 80–100 °C, 230–300 °C, and 390–440 °C. The first stage was attributed to the evaporation of water from the hydrogel, and the water content was 55%, according to calculation. Upon heating to 230 °C, the second stage of mass loss happened, due to the condensation of adjacent amide groups on the AM units, forming imide bonds and releasing NH_3_, and the decarboxylation of carboxyl groups in the AA units, releasing CO_2_. The third mass loss stage corresponded to the breakdown of the polymer backbone. As the cross-linker was replaced with PEGDA, the resulting TG and DTG curves exhibited the same trend, indicating that the incorporation of the low-coverage polyrotaxane did not compromise the thermal stability of the hydrogel (Fig. 1c and d). Swelling experiments demonstrated that the dried hydrogel could absorb approximately 90 times its dry weight in water and remain stable in water for at least 14 days without dissolving. To further assess the conductivity of the hydrogel, electrochemical impedance spectroscopy was used to measure resistance, from which a conductivity of 0.65 S m^−1^ was calculated, indicating good electrical conductivity (Fig. S18).

Rheological tests on slide-ring hydrogels with various rotaxane contents, together with the hydrogel cross-linked with PEGDA, were carried out to investigate the mechanical properties of slide-ring supramolecular hydrogels. In the stress scanning curves of these hydrogels, as the stress increased from 0.1% to 1000%, the storage modulus (*G*′) was initially higher than the loss modulus (*G*″) and then gradually approached it, indicating progressive disruption of the hydrogel network with increasing stress. The loss factor (tan *δ*), defined as the ratio of *G*″ to *G*′, exhibited a relatively low value at low stresses, reflecting the elastic solid state of the material, and as stress increased, the rise in tan *δ* indicated the enhanced viscous flow response. In the frequency scanning curves, as the frequency increased from 0.1 rad s^−1^ to 100 rad s^−1^, both *G*′ and *G*″ gradually increased and remained roughly parallel, with *G*′ consistently higher than *G*″, demonstrating the stability of the hydrogels within this range (Fig. 1e, f and S19). Notably, differences in rotaxane content did not significantly affect the rheological properties of the hydrogels (Fig. S20). However, the storage modulus of the supramolecular slide-ring hydrogels was significantly higher than that of the PEGDA-cross-linked hydrogel, confirming that the low-coverage polyrotaxane cross-linker enhanced the mechanical strength of the hydrogel. The self-healing capability of the slide-ring hydrogel was evaluated by measuring the rheological recovery at a fixed frequency of 1 Hz with stepwise strain (strain = 1%–2000%–1%–2000%) at 25 °C. At a high strain of 2000%, the hydrogel network was disrupted, resulting in a loss of its mechanical strength (*G*″ > *G*′). Meanwhile, as the strain was reduced back to 1%, the reversibility of hydrogen bonds promoted the recovery of the hydrogel network from the disrupted viscous state to the elastic solid state, demonstrating self-healing behaviour of the slide-ring hydrogel (Fig. 1g).

**Fig. 2:**
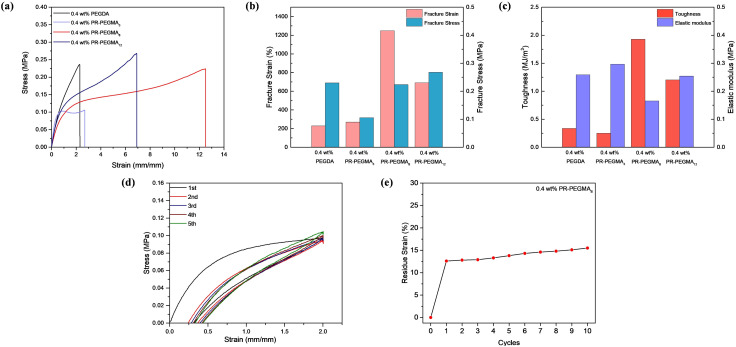
(a) Tensile stress–strain curves of the PEGDA/PEDOT:PSS/AM/AA hydrogel (black) and PR-PEGMA_*x*_/PEDOT:PSS/AM/AA hydrogels (purple, *x* = 3; red, *x* = 8; dark blue, *x* = 12). (b) The fracture stresses and the fracture strains and (c) the tensile toughness and the elastic moduli of the PEGDA/PEDOT:PSS/AM/AA hydrogel and PR-PEGMA_*x*_/PEDOT:PSS/AM/AA hydrogels (*x* = 3, 8, 12). (d) Tensile loading–unloading curves of the PR-PEGMA_8_/PEDOT:PSS/AM/AA hydrogel. (e) Residual strain as a function of cycle times at a strain of 200% for the PR-PEGMA_8_/PEDOT:PSS/AM/AA hydrogel.

Tensile tests were conducted on slide-ring hydrogels with varying rotaxane contents and different numbers of PEGMA side chains to quantitatively analyze the effects of rotaxane content and side-chain density on mechanical properties, and hydrogels cross-linked without rotaxane structures and with high-coverage polyrotaxane were tested as controlled groups. Among the slide-ring hydrogels prepared with low-coverage polyrotaxane cross-linkers bearing different numbers of side chains, as the number of PEGMA side chains increased from 3 to 12, the fracture stress gradually increased, while the fracture strain and toughness first rose and then decreased. The elastic modulus first decreased and then increased, indicating that either too many or too few side chains could compromise the flexibility of the slide-ring hydrogels (Fig. 2a–c). It is worth noting that the low-coverage polyrotaxane hydrogels exhibited strain-hardening behaviour, as indicated by the stress–strain diagrams showing an increasing slope after the yield region, accompanied by a significant rise in the elastic modulus, suggesting that the samples became stiffer upon deformation. Compared to the PEGDA cross-linked hydrogel, the polyrotaxane hydrogel showed lower fracture strain but higher fracture stress and elastic modulus, which could be attributed to the sliding cross-links provided by the polyrotaxane structure, where the threaded cyclodextrins could move reversibly along the PEG chains, effectively dissipating energy during stretching.^35^ As the content of the low-coverage polyrotaxane cross-linker was increased from 0.4 wt% to 1.6 wt%, the fracture strain, elastic modulus, and toughness decreased from 1248.46% to 707.5%, 0.165 MPa to 0.102 MPa, and 1.93 MJ m^−3^ to 1.05 MJ m^−3^, respectively, while the fracture stress first increased and then decreased (Fig. S21), attributed to the increased cross-linking density and number of slide-rings resulting from higher cross-linker content, which enhances flexibility but markedly reduces toughness. In contrast, hydrogels cross-linked with high-coverage polyrotaxane showed nearly unchanged fracture stress but decreased fracture strain and toughness (Fig. S22), suggesting that the high-coverage restricts sliding behaviour. Meanwhile, low-coverage polyrotaxane retained active sliding, which improved both flexibility and achievable strain. The hydrogel prepared with 0.4 wt% PR-PEGMA_8_ exhibited optimal mechanical properties with a fracture strain of 1248%, a fracture stress of 0.224 MPa, an elastic modulus of 0.165 MPa, and toughness of 1.93 MJ m^−3^. Variations in tensile properties among these samples verified that low-coverage polyrotaxane significantly enhanced the flexibility and fracture strain of the hydrogels. Therefore, the PR-PEGMA_8_/PEDOT:PSS/AM/AA hydrogel was selected for subsequent characterization.

The fatigue resistance of the PEGMA_8_/PEDOT:PSS/AM/AA hydrogel was characterized through cyclic tensile testing at 200% strain. With a cross-linker content of 0.4 wt%, the hydrogel exhibited a large hysteresis area in the first stretching cycle, but in subsequent cycles, the hydrogel could almost fully recover, and the hysteresis area greatly reduced (Fig. 2d), attributed to the presence of unstable structures within the hydrogel such as locally short polymer chains or a small amount of uncapped polypseudorotaxane structure, which were irreversibly disrupted during the first cycle. In contrast, other types of cross-linked networks showed inferior fatigue resistance (Fig. S23). The cyclic residual strain was evaluated by subjecting the hydrogel to 10 consecutive cycles under a strain of 200%. The PR-PEGMA_8_/PEDOT:PSS/AM/AA hydrogel showed a residual strain of 12.6% after the first cycle, which remained largely stable in the subsequent cycles and did not exceed 16% after 10 cycles (Fig. 2e and S24).

**Fig. 3:**
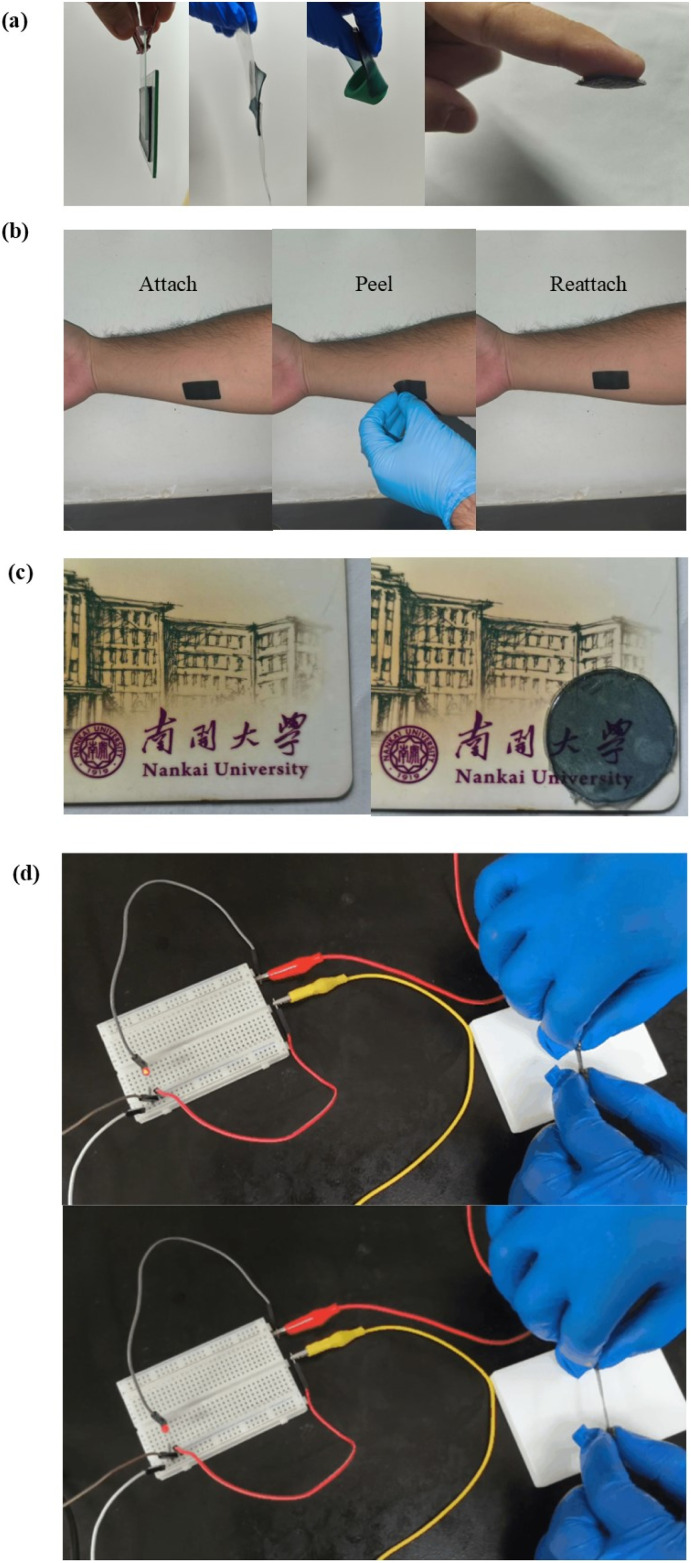
(a) The hydrogel exhibiting universal adhesiveness on material surfaces of glass, plastics, rubber and human skin. (b) The hydrogel repeatedly attaching to the surface of human skin. (c) Optical photo of a transparent hydrogel covering the ID card of Nankai University. (d) Hydrogel conducting electricity to brighten a bulb, and the LED light becoming darker when the hydrogel is stretched.

The low-coverage slide-ring hydrogel exhibited good formability and processability. It is widely known that the preparation of a hydrogel into a wearable sensor needs to meet several conditions, such as adhesion and transparency. The hydrogel could adhere to various surfaces such as glass, plastic, rubber, and human skin (Fig. 3a and S25). It is of great importance that the hydrogel not only attached effectively to skin but could also be reattached after removal and could be detached without leaving any residue or allergic reactions (Fig. 3b). Fonts and colours covered by the hydrogel remained clearly visible, confirming that the material retained good optical transparency, despite its blue-black appearance (Fig. 3c). When integrated into a closed circuit, the hydrogel could effectively conduct current, illuminating a connected LED. As tensile strain was applied to the hydrogel, the brightness of the light dimmed progressively with increasing strain, and as the hydrogel was recovered, the light was illuminated again (Fig. 3d). Owing to the favourable conductivity and stretchability, the low-coverage slide-ring hydrogel was suitable for applications in flexible wearable electronic devices, enabling real-time monitoring of dynamic human motions such as joint movement.

**Fig. 4:**
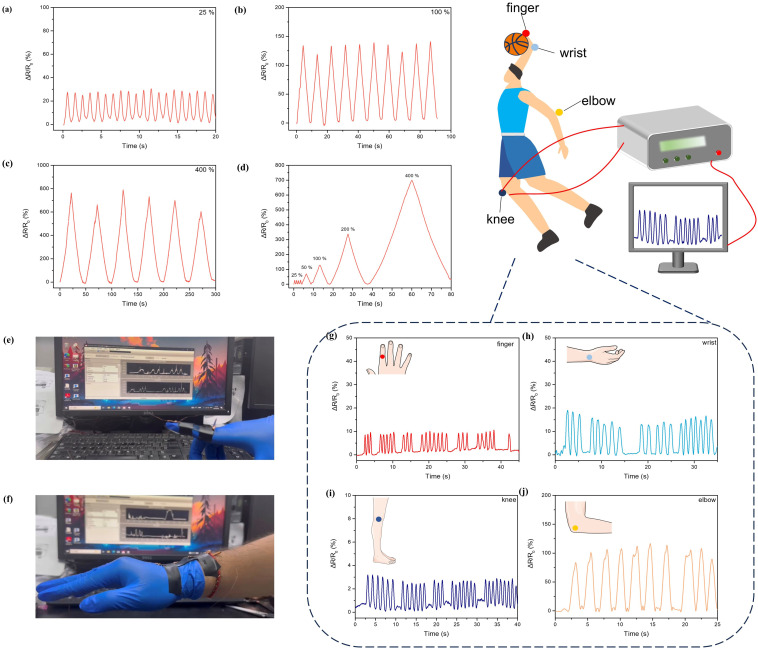
Δ*R*/*R*_0_ curves of the hydrogel during the stretching-recovery cycle when the tensile strain is (a) 25%, (b) 100% and (c) 400%. (d) Δ*R*/*R*_0_ curves of the hydrogel under different tensile strains (PR-PEGMA_8_/PEDOT:PSS/AM/AA hydrogel). (e and f) The image of sensing application of a strain sensor. Real-time signal of the (g) finger, (h) wrist, (i) knee and (j) elbow movement.

Resistive sensing and triboelectric sensing represent two major frontiers in flexible wearable sensing electronic devices. The low-coverage slide-ring hydrogel was connected to an electrometer *via* copper tape and wires, to evaluate its potential as a resistive sensor by investigating the relative resistance change under different tensile strains using an external power source. As the strain increased from 25% to 400%, the relative resistance change of the hydrogel progressively rose from 30% to 700% (Fig. 4a–d, S26a and b). During multiple cyclic tests, the hydrogel recovered its original shape after each release, with resistance returning to the initial value. Moreover, the hydrogel exhibited a gauge factor (GF) of 1.916, meeting the requirements for monitoring large-amplitude, low-frequency human motions (Fig. S26c), indicating that the hydrogel-based resistive sensor possesses high strain sensitivity and reliable electrical stability over a wide strain range, because the cross-linked network effectively dissipated energy induced by stretching, enabling regular and predictable resistance variation upon deformation. By attaching to human skin, the sensor could effectively detect body movements, and by monitoring the relative resistance change (Δ*R*/*R*_0_) during motions of the finger, wrist, elbow, and knee, the hydrogel-based resistive sensor exhibited distinct and real-time response patterns under different types and intensities of movement. In particular, varying bending angles of the elbow led to clearly differentiated levels of resistance change (Fig. 4e–j). When the joints returned to their initial positions, the resistance changes basically reverted to baseline values, which confirmed the high strain sensitivity of the hydrogel-based resistive sensor and demonstrated its capability to reliably capture steady-state, large-magnitude motion signals.

**Fig. 5:**
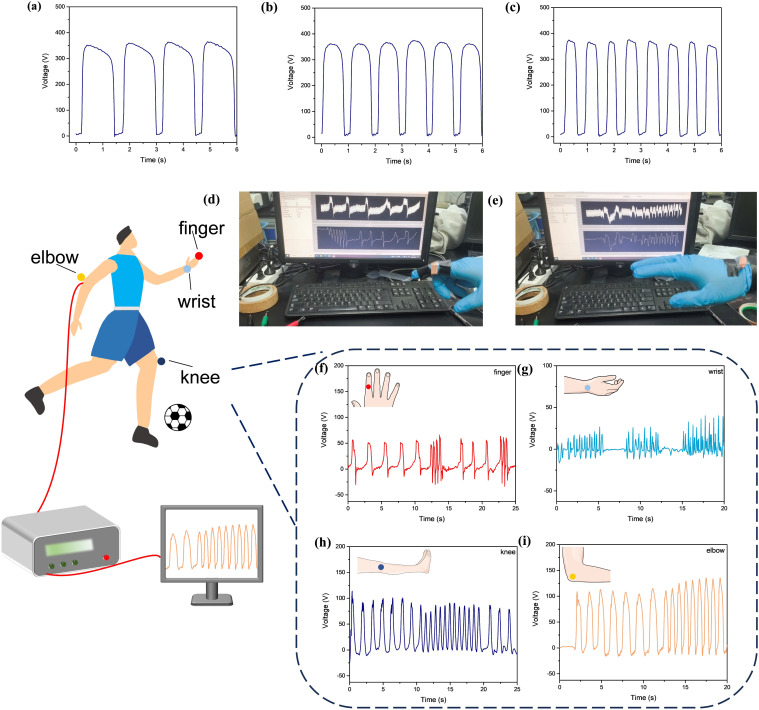
*V*_OC_ of a PDMS-VHB single electrode TENG with a contact separation frequency of (a) 0.67 Hz, (b) 1.0 Hz and (c) 1.4 Hz. (d and e) The image of sensing application of a TENG sensor. Real-time signal of the (f) finger, (g) wrist, (h) knee and (i) elbow movement.

The low-coverage slide-ring hydrogel was encapsulated between two layers of polydimethylsiloxane (PDMS) films and connected to an electrometer *via* copper tape and wires, constructing a stretchable single-electrode triboelectric nanogenerator (TENG) with a three-layer structure, which was developed into a self-powered triboelectric sensing system. The performance of the single-electrode TENG was evaluated using the contact-separation method, with VHB selected as the counter dielectric material. When the contact-separation frequency changed to 0.67, 1, and 1.4 Hz, the open-circuit voltage (*V*_OC_) and short-circuit charge (*Q*_SC_) remained stable at 350 V and 8.0 nC, respectively, while the short-circuit current (*I*_SC_) increased from 0.17 µA to 0.3 µA with increasing frequency (Fig. 5a–c and S27). Rapid contact-separation generated higher electrical signal frequencies; similarly, slower movement produced lower frequencies, enabling the TENG-based sensor to effectively reflect motion speed without external power. The stretchable TENG was attached to the finger, wrist, elbow, and knee as a self-powered sensor for monitoring human motion (Fig. 5d, e). With joint bending or extension, triboelectrification induced significant voltage output variations, with the monitored voltage amplitude increasing alongside the range of joint motion, and moreover, the output voltage frequency of the TENG varied with the speed of joint movement, allowing the monitoring of motion frequency (Fig. 5f–i). It is worth noting that the hydrogel remained intact after testing, demonstrating its stability under different conditions. These results confirmed the high responsiveness of the hydrogel-based triboelectric sensor in capturing dynamic, high-frequency motion signals.

## Conclusions

In conclusion, we successfully developed a novel highly elastic and self-generating supramolecular low-coverage slide-ring hydrogel through the copolymerization of acrylamide (AM) and acrylic acid (AA) with a low-coverage polyrotaxane cross-linker, which was fabricated by threading a controlled number of cyclodextrins onto the poly(ethylene glycol) (PEG) chain. The low-coverage polyrotaxane structure is the key to significantly enhanced mechanical properties of the hydrogel, while the incorporation of poly(3,4-ethylenedioxythiophene):poly(styrenesulfonate) (PEDOT:PSS) provided an excellent conductivity of 0.65 S m^−1^. When applied as a resistive sensor, the hydrogel exhibited a GF of 1.916, meeting the requirements for monitoring large-scale and low-frequency human motions. As an electrode for a triboelectric nanogenerator (TENG), the hydrogel can generate an open-circuit voltage on the order of 10^2^–10^3^ V and a short-circuit current on the µA scale, ensuring that it is suitable for self-generating triboelectric sensing. We believe that this low-coverage cyclodextrin slide-ring hydrogel is promising for flexible dual-modal human-to-computer information conversion, thereby broadening the application scope for supramolecular slide-ring hydrogel-based flexible materials.

## Author contributions

The manuscript was written through contributions of all authors. Conceptualization: X. Pan, Y. Chen, Y. Liu; methodology: X. Pan, X.-Y. Yu, J.-L. Yue; investigation: X. Pan, B. Wang, X.-Y. Yu, C. Zhang; visualization: X. Pan, X.-Y. Yu, X. Zhang; supervision: Y.-M. Zhang, Y. Chen, Y. Liu; writing – original draft: X. Pan, X.-Y. Yu; writing – review & editing: Y.-M. Zhang, Y. Chen, Y. Liu.

## Conflicts of interest

There are no conflicts to declare.

## Supplementary Material

SC-OLF-D6SC00045B-s001

## Data Availability

The data that support the findings of this study are available in the supplementary information (SI). Supplementary information: synthesis and characterization of compounds, ^1^H NMR spectra, 2D NOESY, FT-IR spectra, XPS spectra, SEM images, rheological tests, electrochemical AC impedance spectra, tensile stress–strain curves, tensile toughness, elastic modulus, residue strain, image of the appearance of the hydrogel, Δ*R*/*R*_0_ curve, short-circuit charge (*Q*_SC_), and short-circuit current (*I*_SC_) of the TENG. See DOI: https://doi.org/10.1039/d6sc00045b.
