# Supplementary material for: Stretchable cyclodextrin slide-ring copolymer self-generating hydrogels for high-performance flexible electronics
Source: Chem Sci. 2026 Mar 25;17(20):10104–12. doi: 10.1039/d6sc00045b (PMC13063983; doi:10.1039/d6sc00045b)
Supplement: SC-017-D6SC00045B-s001 [file SC-017-D6SC00045B-s001.pdf]

Supplementary Information (SI) for Chemical Science.
This journal is © The Royal Society of Chemistry 2026

Supplementary Information

Stretchable Cyclodextrin Slide-Ring Copolymer Self-Generating Hydrogel for High-Performance Flexible Electronics

Xu Pan,[a] Bo Wang,[a,b] Xiao-Yong Yu,[a] Jin-Long Yue,[a] Xu Zhang,[a] Chen Zhang,[a] Ying-Ming Zhang,[a] Yong Chen,[a,] and Yu Liu[a,*]*

a College of Chemistry, State Key Laboratory of Elemento-Organic Chemistry, Nankai University, Tianjin 300071, P. R. China.

b Shandong Binzhou Zhiyuan Biotechnology Co., Ltd, Shandong 256500, P. R. China

*Corresponding author. Email: chenyong@nankai.edu.cn; yuliu@nankai.edu.cn

Instrumentation and methods

All ^{1}H NMR spectra and 2D NOESY spectra were recorded on a Bruker AV400 spectrometer. The microscopic morphology of the hydrogel was obtained on a JSM-7500F cold field emission scanning electron microscope. The infrared spectrum was recorded on TENSOR II Fourier transform infrared spectrometer. Disc-shaped samples with thicknesses of 2 mm and diameters of 20 mm were used to perform the rheological tests on an AR 2000ex (TA Instrument) system at 25 °C. Tensile tests were conducted on MTS CMT4000 series universal testing machine. Uniaxial tests and cyclic loading–unloading tests of tensile measurements were conducted with the stretching rate of 50 mm/min at room temperature, and samples for tensile measurements were cut into dumbbell-shaped slices. $\Delta R/R_0$ of the hydrogel under different tensile strains, open circuit voltage (V_{OC}), short-circuit charge (Q_{SC}) and short-circuit current (I_{SC}) of TENG were tested by the Keithley electrometer 6514. Electrochemical AC impedance was conducted on an electrochemical workstation by placing the hydrogel (5×4×1 mm^3) between two platinum electrode plates (10×5 mm^2) for testing. The frequency range was set from 1 MHz to 0.01 Hz with an amplitude of 50 mV.

Synthesis of PR-PEGMA$_x$. PR-PEGMA$_x$ was synthesized according to the method reported in previous literature.[1,2] Polyethylene glycol (PEG$_{10000}$, 10 g) was dissolved in anhydrous pyridine (50 mL) with 1,8-diazabicyclo[5.4.0]undec-7-ene (DBU, 250 µL) and 2,2-dimethylsuccinic anhydride (6.0 mL). The solution was stirred at 60 °C overnight under nitrogen atmosphere and then dropped into deionized water (200 mL) with the pH adjusted to 1-2. The product was extracted with dichloromethane (DCM) and then dried to obtain PEG-S as a white solid.

PEG-S (1.5 g) was dissolved in deionized water (10 mL) with a solution of α–cyclodextrin (α-CD, 6.00 g/40 mL). The mixed solution was stirred for 30 minutes, stored in a refrigerator for two days, and then was freeze-dried to obtain a white powder containing pseudopolyrotaxane.

A solution of (benzotriazol-1-yloxy)tris(dimethylamino)phosphonium

hexafluorophosphate (BOP, 570 mg), 1-adamantanamine (195 mg), and N,N-Diisopropylethylamine (DIPEA, 240 μL) in anhydrous acetonitrile (75 mL) was mixed with the aforementioned pseudopolyrotaxane under nitrogen atmosphere and stirred at room temperature for 48 hours. The resultant suspension was centrifuged to remove the supernatant. The obtained solid was washed with acetonitrile twice, and then the solid was dried before dissolved in dimethyl sulfoxide (DMSO, 50 mL). The solution was purified by dialysis operation and was freeze-dried to obtain PR-5 as a white solid.

Poly(ethylene glycol) methacrylate (PEGMA, 1.80 mL) was added into an anhydrous DCM solution (80 mL) of 1,1'-carbonyldiimidazole (CDI, 0.99 g), and the solution was stirred at room temperature under nitrogen atmosphere for 24 h followed by saline washing (3×100 mL). The organic phase was dried with anhydrous sodium sulfate and concentrated in vacuo to obtain a yellow viscous liquid (PEGMA-CDI).

CD-PR with a certain amount of PEGMA-CDI and 4-(dimethylamino) pyridine (DMAP) were dissolved in anhydrous DMSO, and then the mixture was stirred at 40 °C for 72 h. Phosphate buffered saline (PBS) solution was added and the solution was dialyzed against water, followed by freeze drying to obtain the PR-$PEGMA_x$ as a white solid.

Synthesis of PR-CA-PEGMA. Polyethylene glycol (PEG_{10000}) was dissolved in deionized water (50 mL) with 2,2,6,6-tetramethylpiperidinyl-1-oxide (TEMPO,50 mg) and sodium bromide (0.5 g). Sodium hypochlorite solution (2.5 mL) was dropped into the solution with the pH adjusted to 10-11, followed by the addition of ethanol (5 mL). The product was extracted with dichloromethane (DCM) and then dried to obtain a white solid. After three ethanol dissolution-reprecipitation cycles were underwent, PEG-CA was obtained. The subsequent experimental steps were identical to those described above.

Calculation of tensile toughness. The tensile toughness was determined as the area under the stress-strain curve up to the fracture point.

Calculation of elastic modulus. The elastic modulus was defined as the slope of the stress-strain curve within the linear elastic regime.

Calculation of residue strain. The residual strain was calculated as the permanent extension relative to the original length, measured after the hydrogel was fully recovered after stretching to a predetermined strain.

Fabrication of the resistive sensor. The hydrogel was interfaced with an electrometer using copper tape and wires at both ends, which enabled the sensor to be directly attached to the skin of human body movement monitoring.

Fabrication of the single-electrode TENG and triboelectronic sensor. A mixture of Sylgard 184 base and curing agent (weight ratio of 10:1) is poured into a teflon mold, then heated at 80 °C for 2 hours to prepare a PDMS film. The TENG was then assembled by clamping the hydrogel ogether with two films. Copper tape and copper wire were connected to the hydrogel for electrical connection to the electrometer for joint movement monitoring.

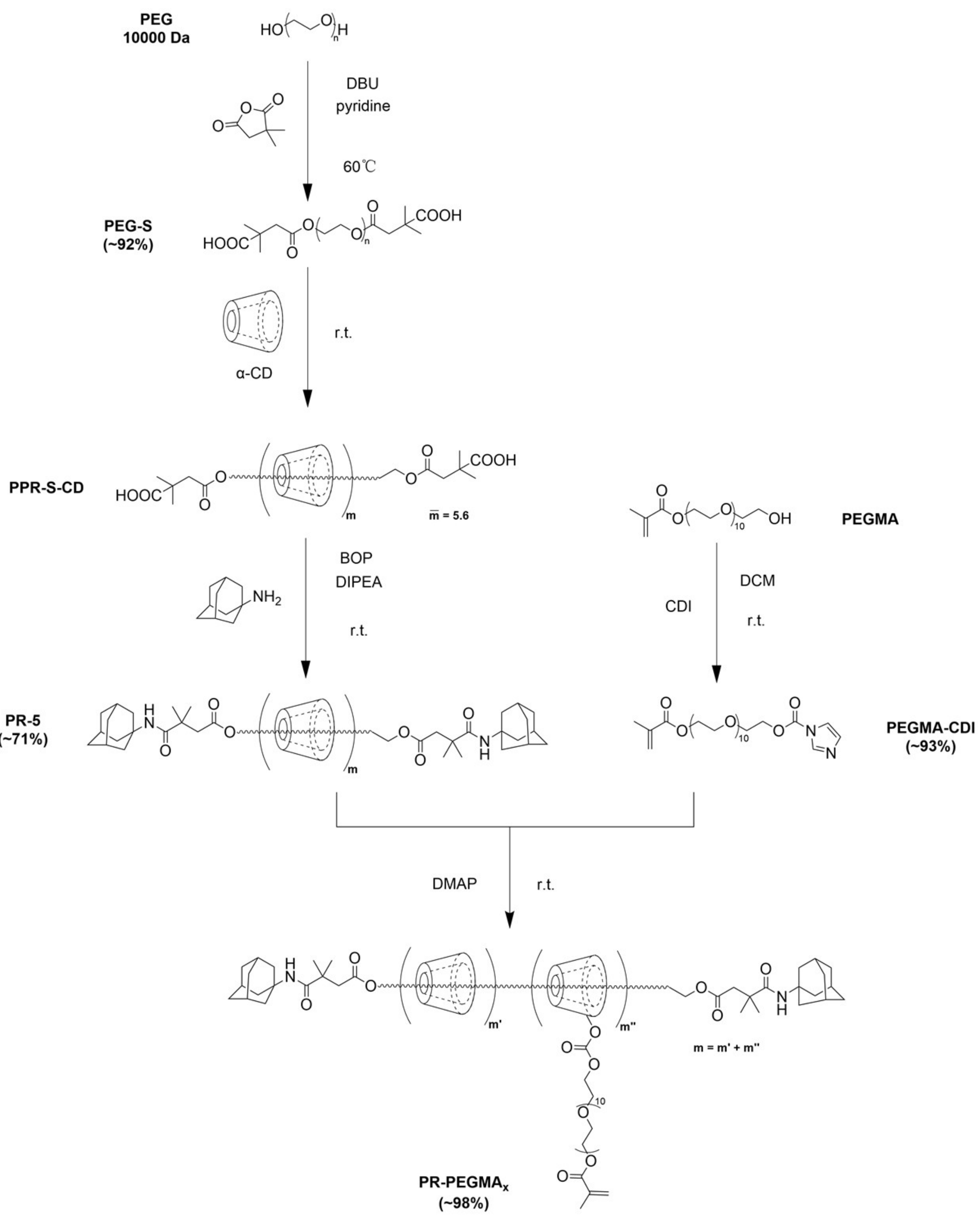

Scheme S1. Synthesis routes of PR-$PEGMA_x$.

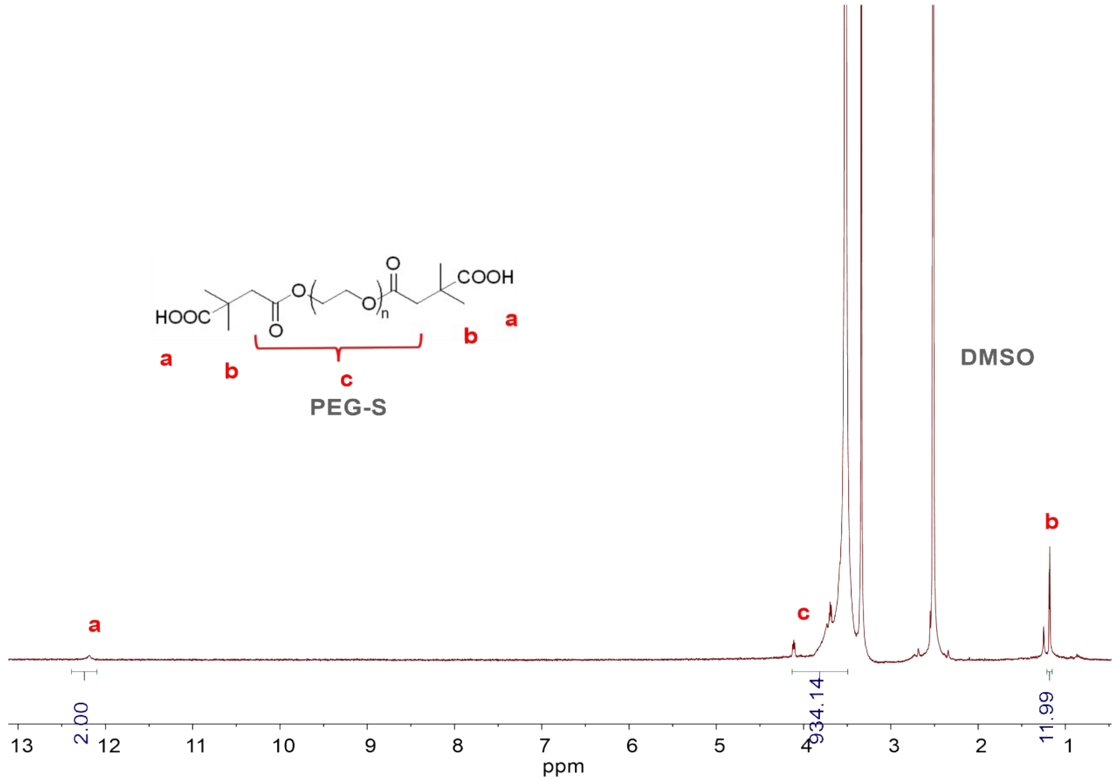

Figure S1. ^{1}H NMR spectrum of PEG-S (400 MHz, DMSO-d_6, 25 °C).

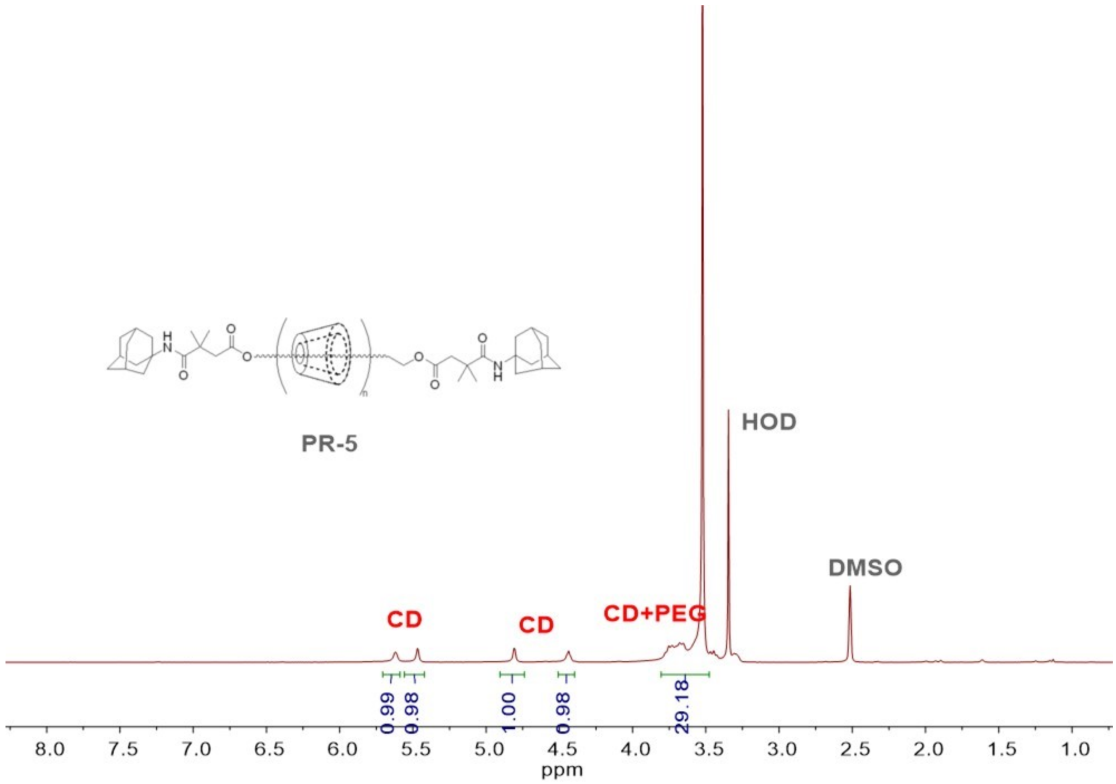

Figure S2. ^{1}H NMR spectrum of PR-5 (400 MHz, DMSO-d_6, 25 °C).

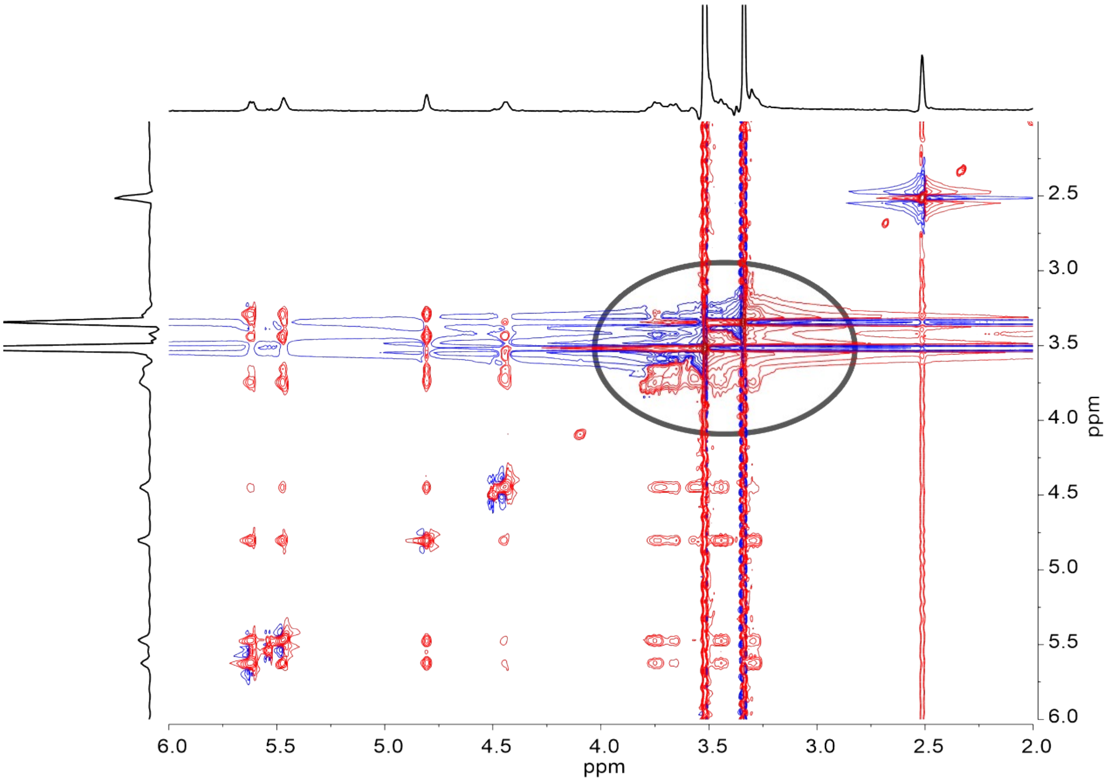

Figure S3. 2D NOESY spectrum of PR-5 (400 MHz, DMSO-d_6, 25 °C).

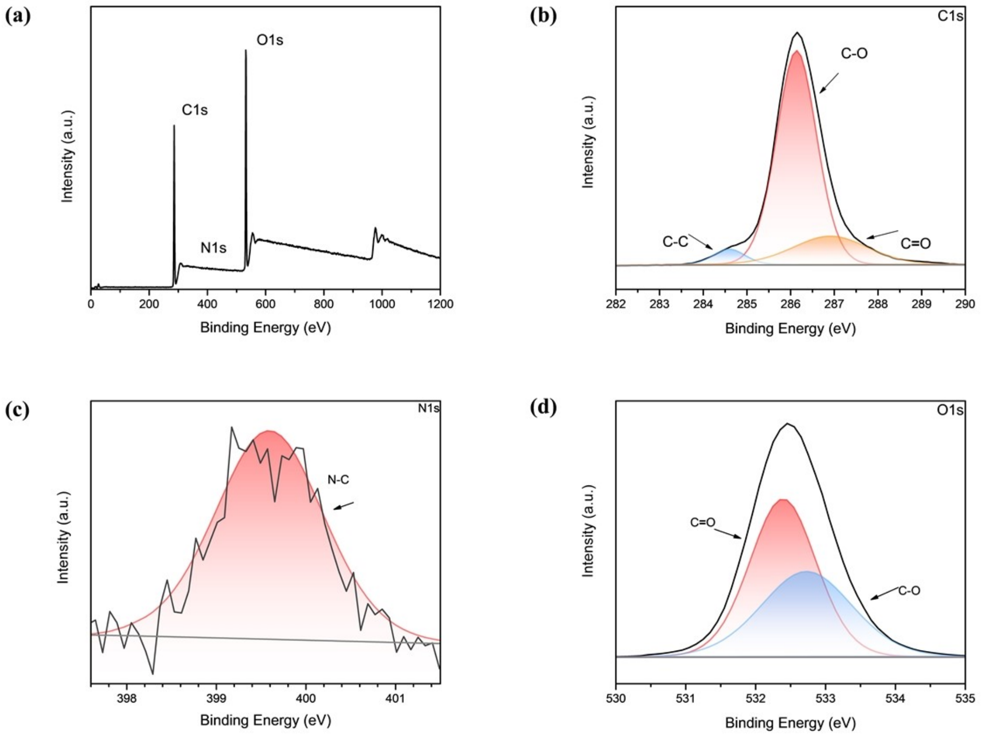

Figure S4. XPS a) Survey, b) C1s, c) N1s, d) O1s spectrum of PR-5.

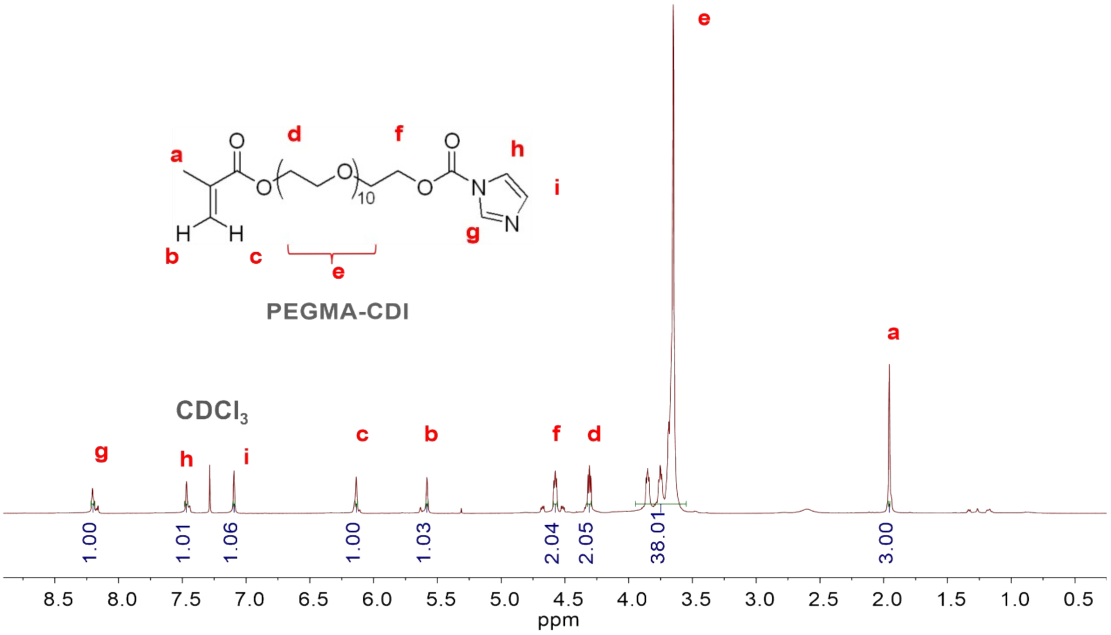

Figure S5. ^{1}H NMR spectrum of PEGMA-CDI (400 MHz, $CDCl_3$, 25 °C).

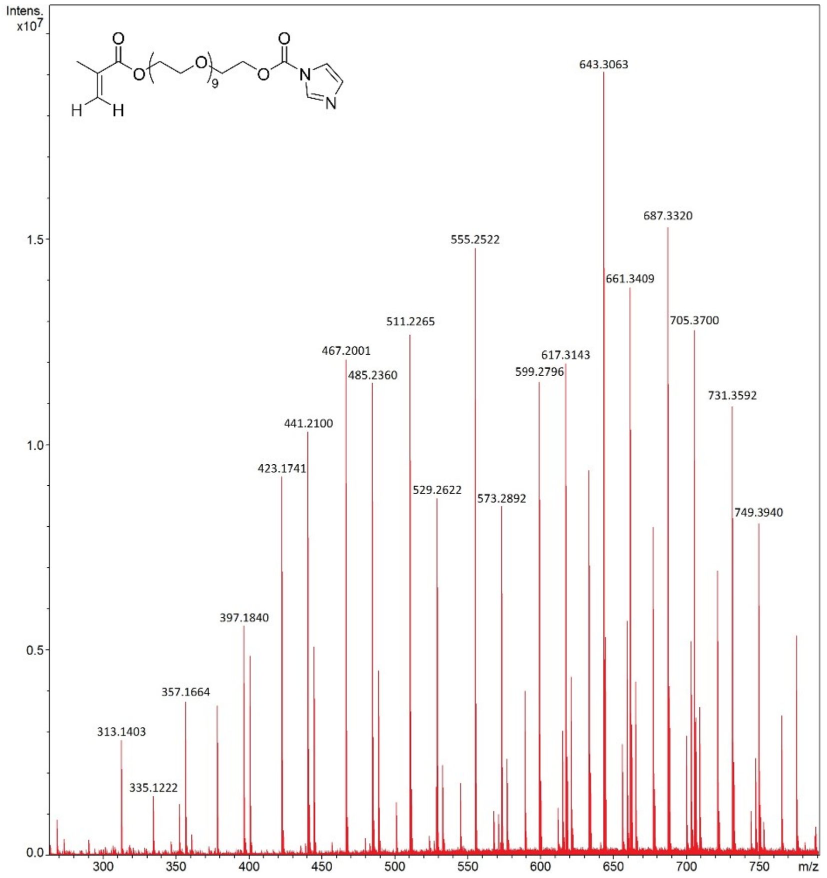

Figure S6. HRMS m/z for PEGMA-CDI calcd. $[M+Na^{+}]^{+}$: 643.6742, found:643.3063.

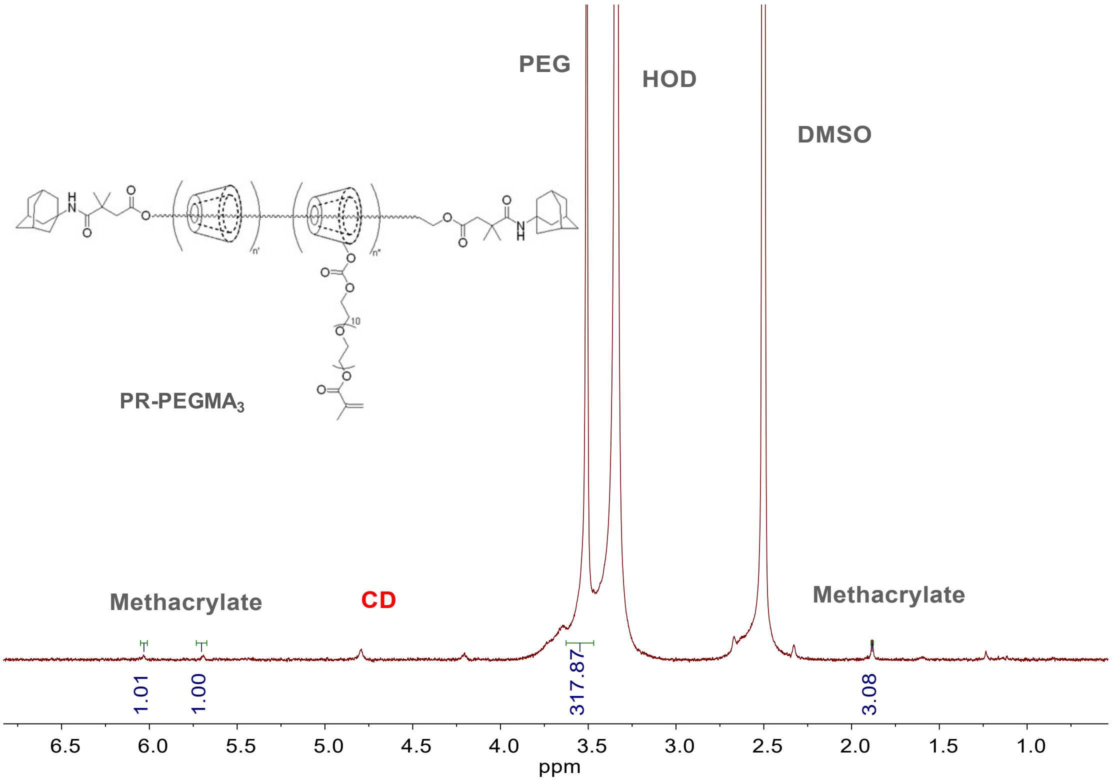

Figure S7. ^{1}H NMR spectrum of PR-PEGMA$_3$ (400 MHz, DMSO-d$_6$, 25 °C).

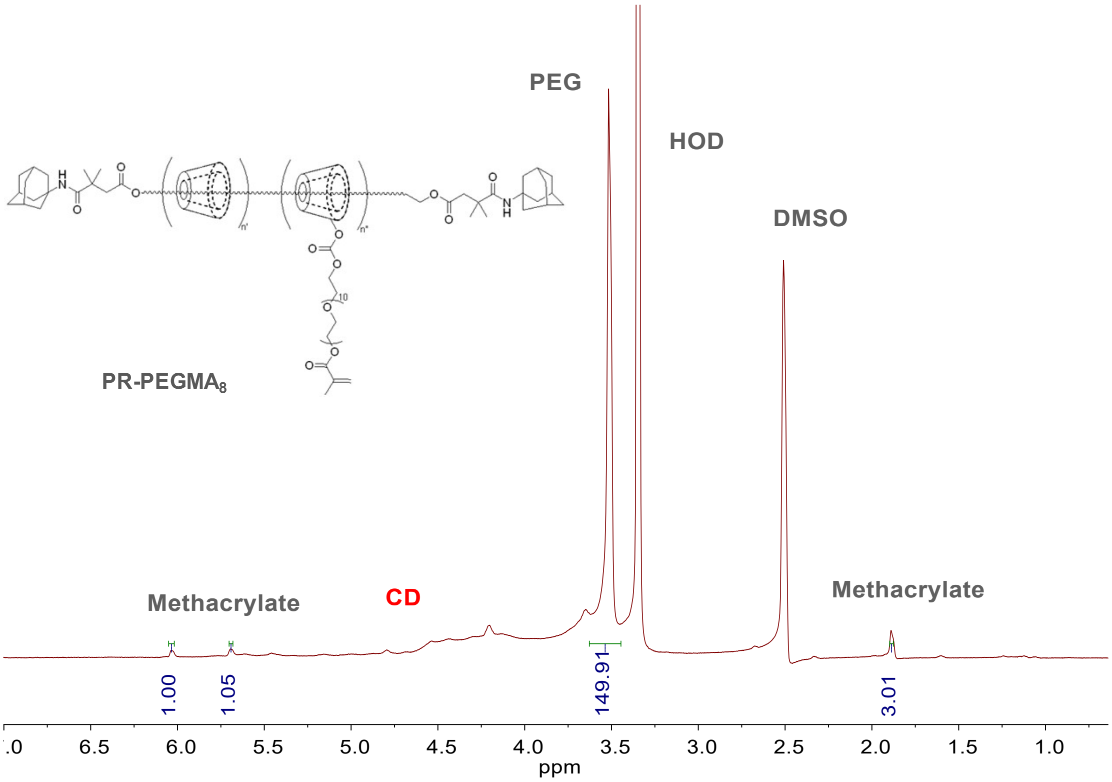

Figure S8. ^{1}H NMR spectrum of PR-PEGMA$_8$ (400 MHz, DMSO-d$_6$, 25 °C).

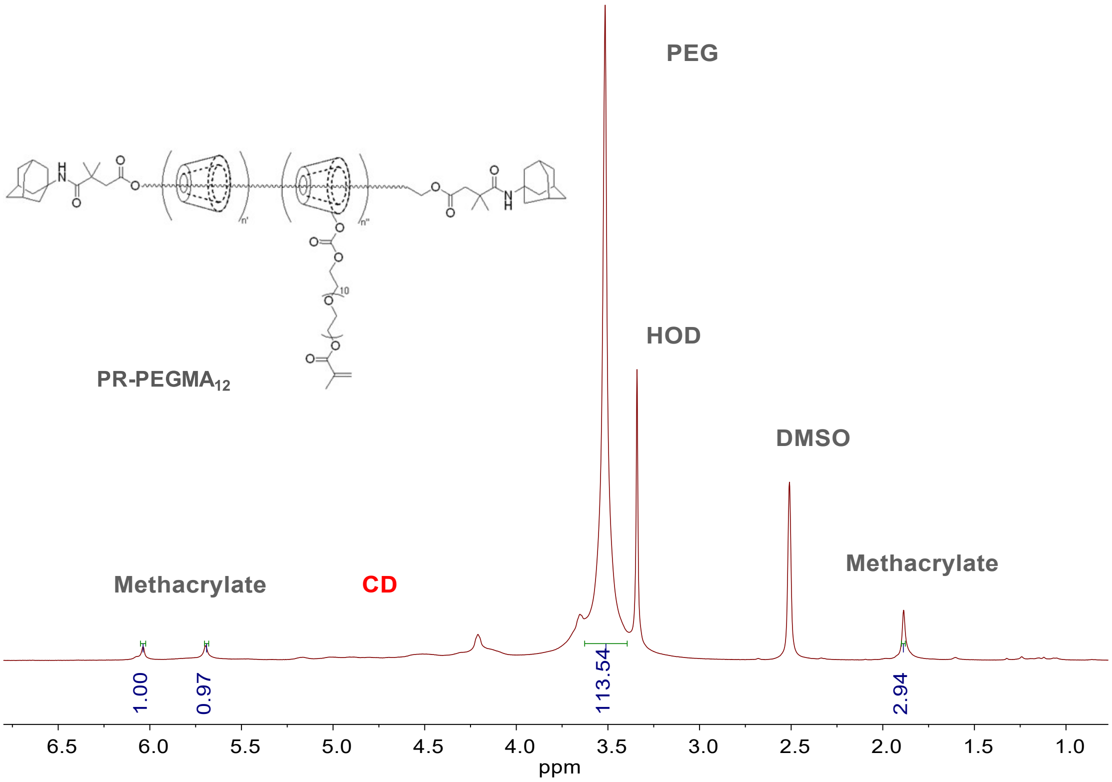

Figure S9. ¹H NMR spectrum of PR-PEGMA$_{12}$ (400 MHz, DMSO-d$_6$, 25 °C).

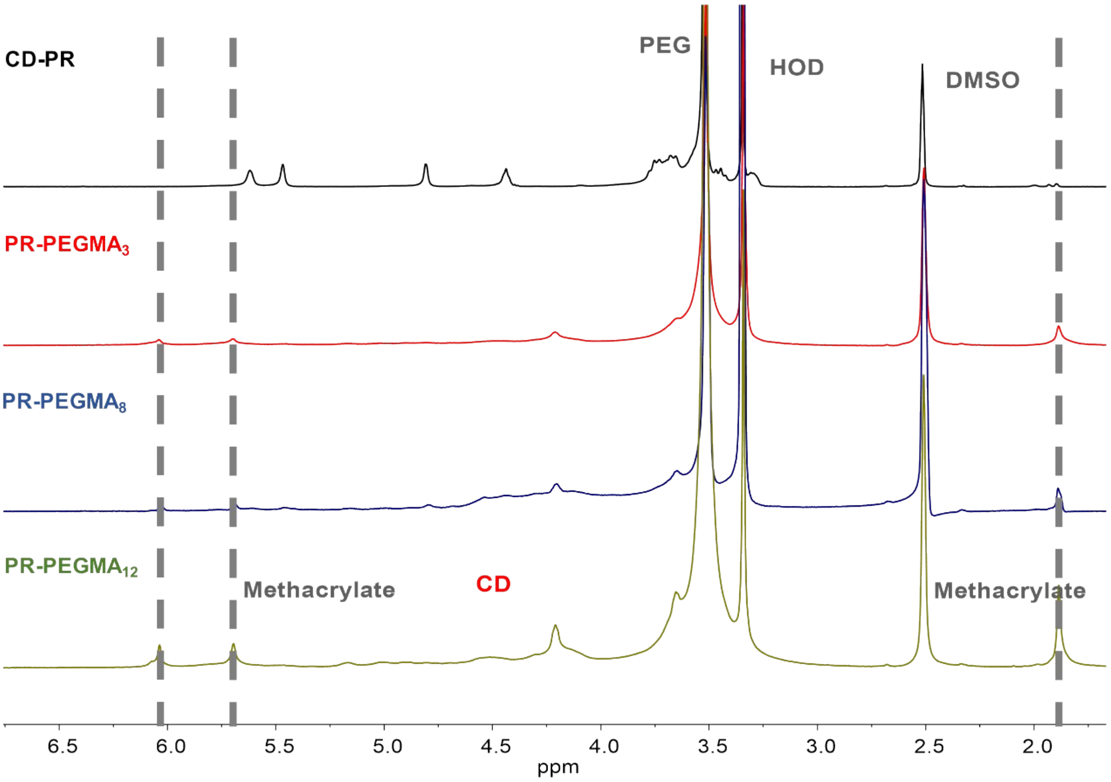

Figure S10. ¹H NMR spectra of PR-PEGMA$_x$ with different number of sidechains in DMSO-d$_6$.

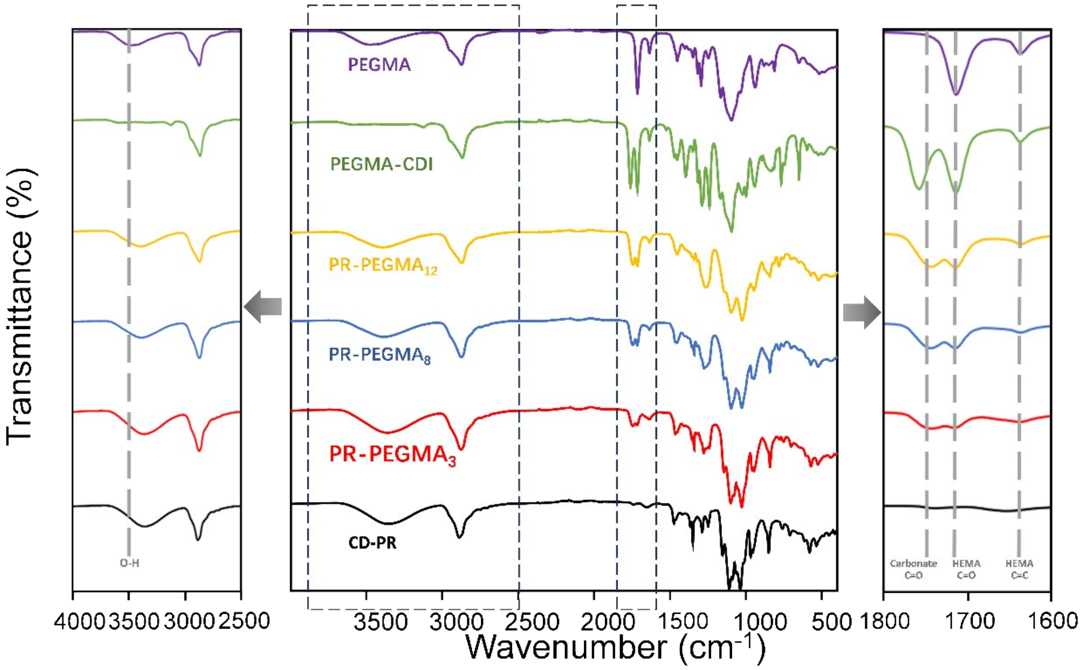

Figure S11. FT-IR spectra of PR-PEGMA$_x$ with different number of sidechains.

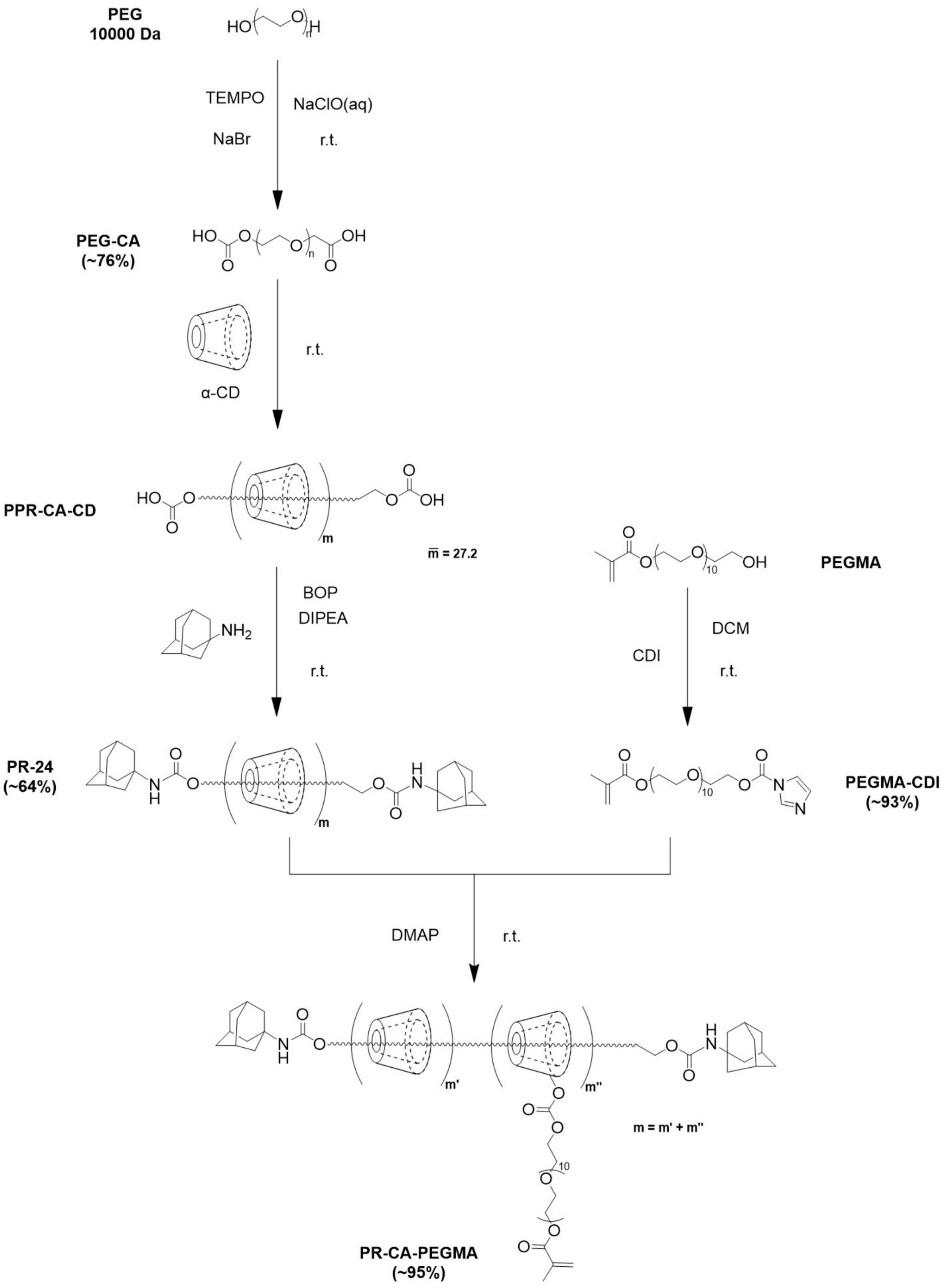

Scheme S2. Synthesis routes of PR-CA-PEGMA.

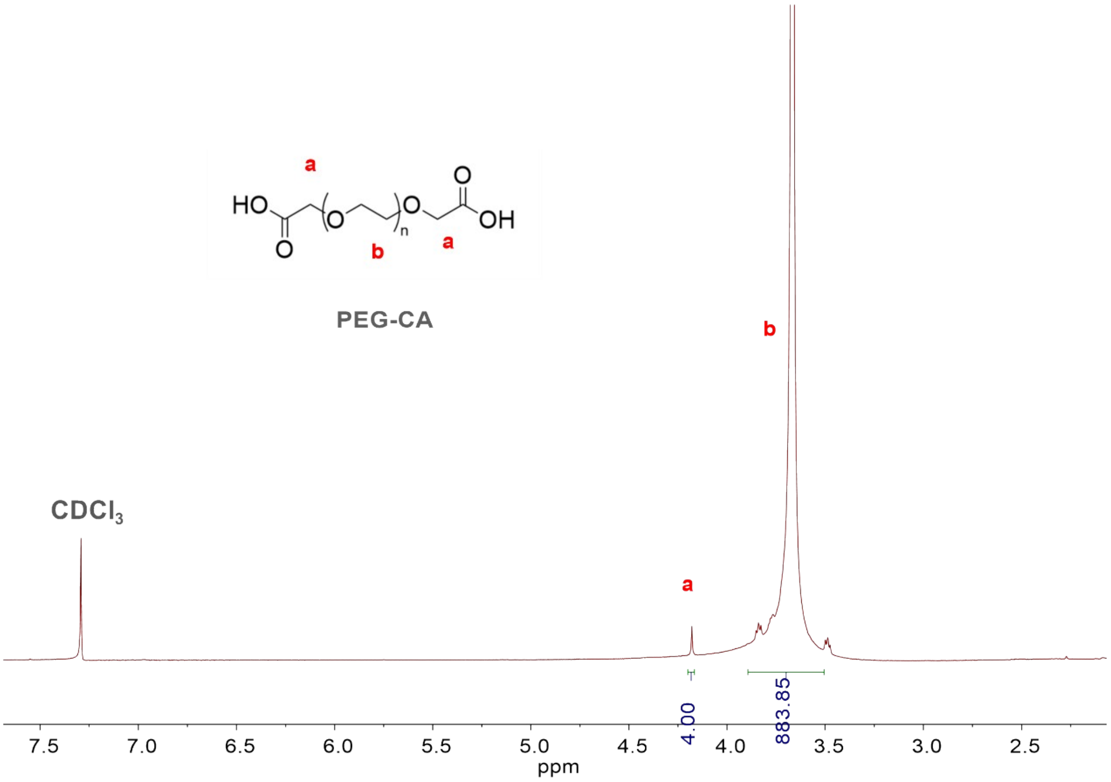

Figure S12. ^{1}H NMR spectrum of PEG-CA (400 MHz, $CDCl_3$, 25 °C).

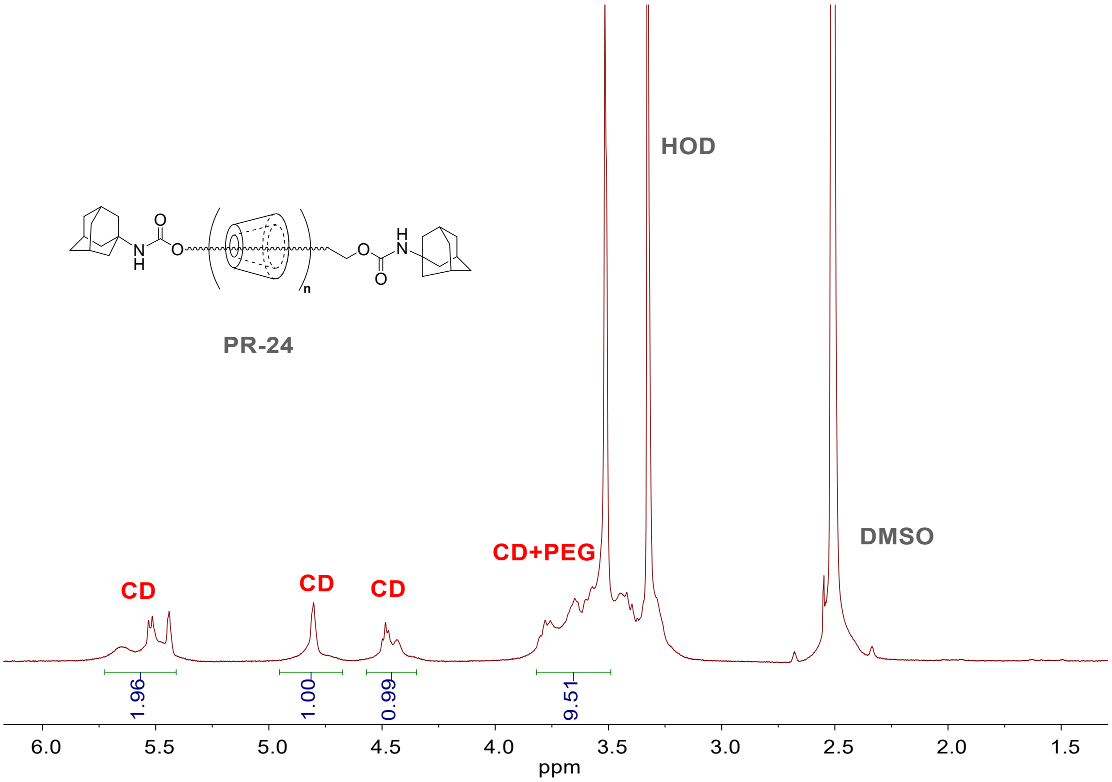

Figure S13. ^{1}H NMR spectrum of PR-24 (400 MHz, DMSO-d_6, 25 °C).

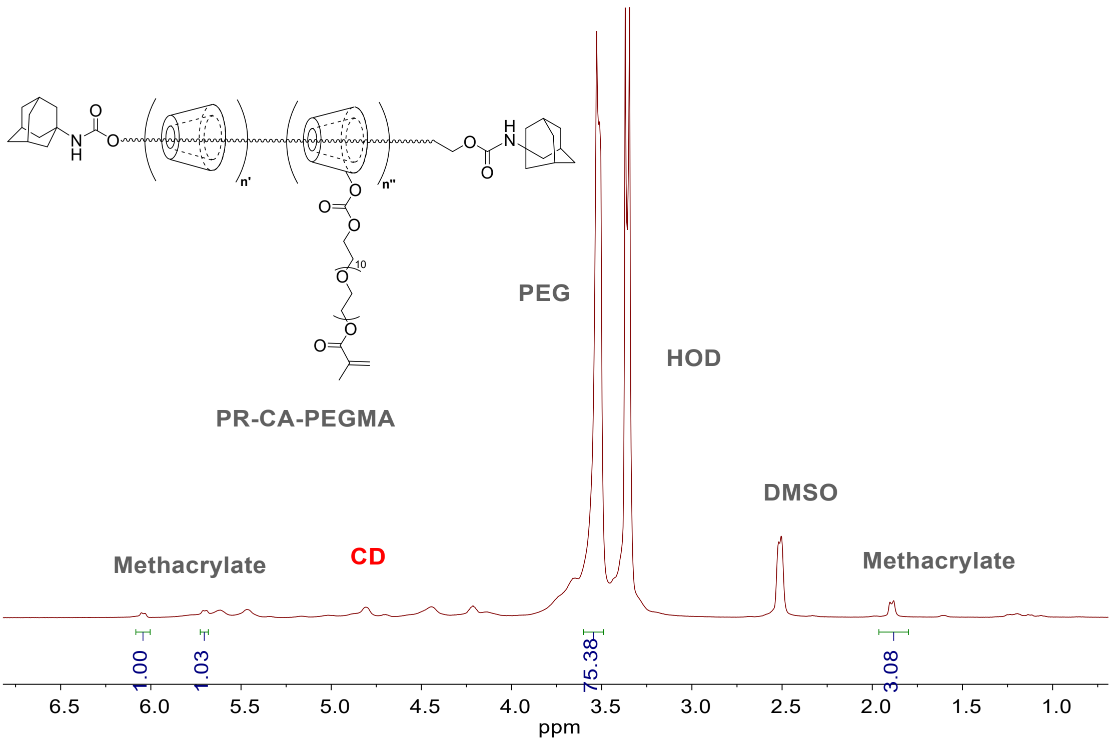

Figure S14. ^{1}H NMR spectrum of PR-CA-PEGMA (400 MHz, DMSO-d_6, 25 °C).

Table S1. Preparation of hydrogels with varying amounts of cross-linker.

AM	AA	cross-linker	PEDOT:PSS	APS	H_2O
3.75 g	0.4 mL	0.4 wt% PEGDA	1.0 mL	45 mg	10 mL
3.75 g	0.4 mL	0.4 wt% PR-CA-PEGMA	1.0 mL	45 mg	10 mL
3.75 g	0.4 mL	0.4 wt% PR-$PEGMA_3$	1.0 mL	45 mg	10 mL
3.75 g	0.4 mL	0.4 wt% PR-$PEGMA_8$	1.0 mL	45 mg	10 mL
3.75 g	0.4 mL	0.4 wt% PR-$PEGMA_{12}$	1.0 mL	45 mg	10 mL
3.75 g	0.4 mL	1.0 wt% PR-$PEGMA_8$	1.0 mL	45 mg	10 mL
3.75 g	0.4 mL	1.6 wt% PR-$PEGMA_8$	1.0 mL	45 mg	10 mL

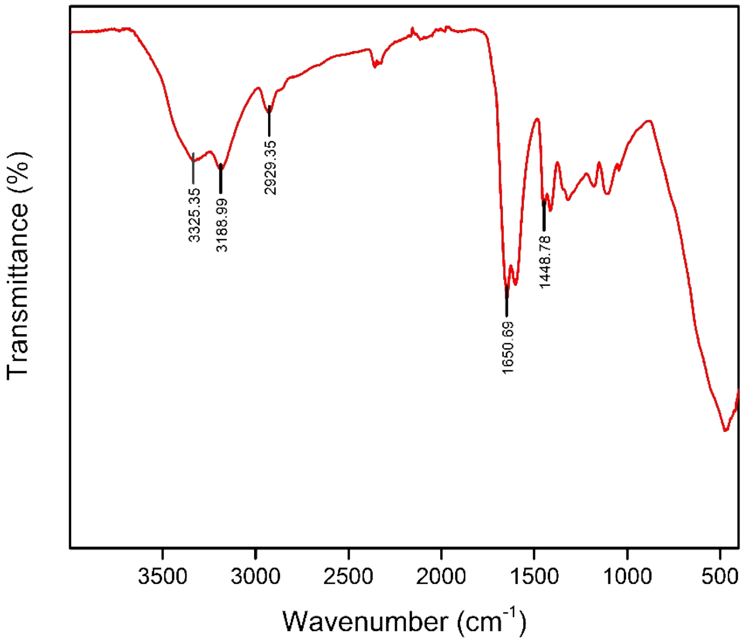

Figure S15. FT-IR spectrum of the frozen-dried PR-PEGMA$_x$/PEDOT:PSS/AM/AA hydrogel.

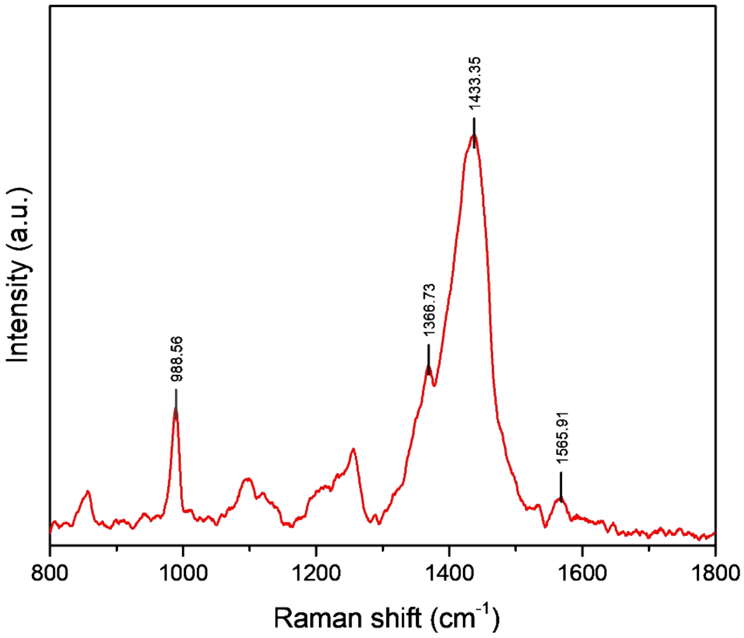

Figure S16. Raman spectrum of the frozen-dried PR-PEGMA$_x$/PEDOT:PSS/AM/AA hydrogel.

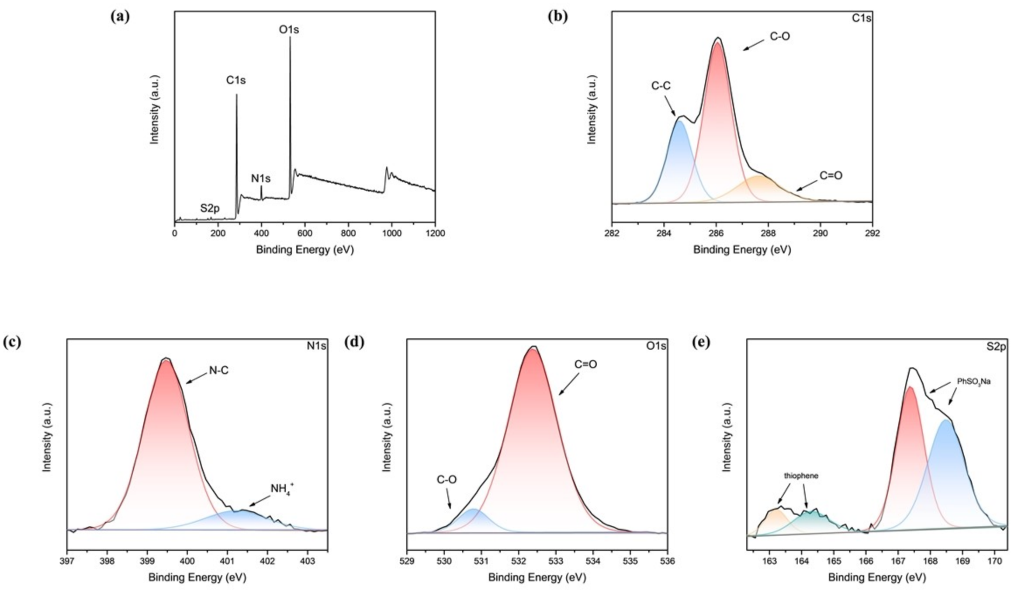

Figure S17. XPS a) Survey, b) C1s, c) N1s, d) O1s, e) S2p spectrum of the frozen dried PR-PEGMA$_X$/PEDOT:PSS/AM/AA hydrogel.

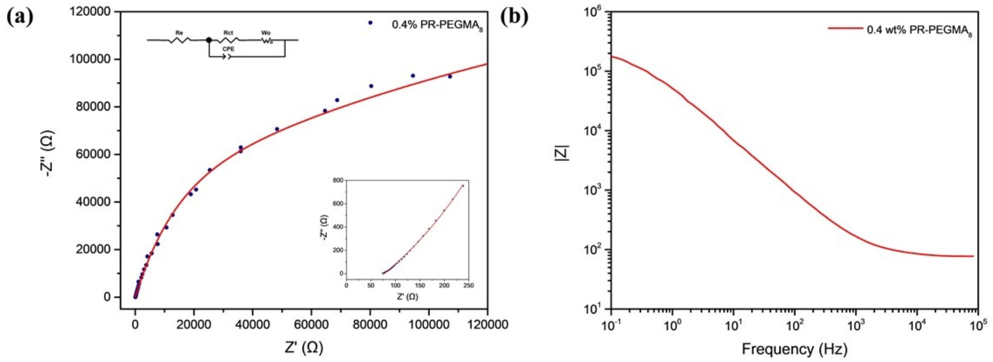

Figure S18. The impedance spectrum a) Nyquist plot, b) Bode plot, of PR-PEGMA$_8$/PEDOT:PSS/AM/AA hydrogel.

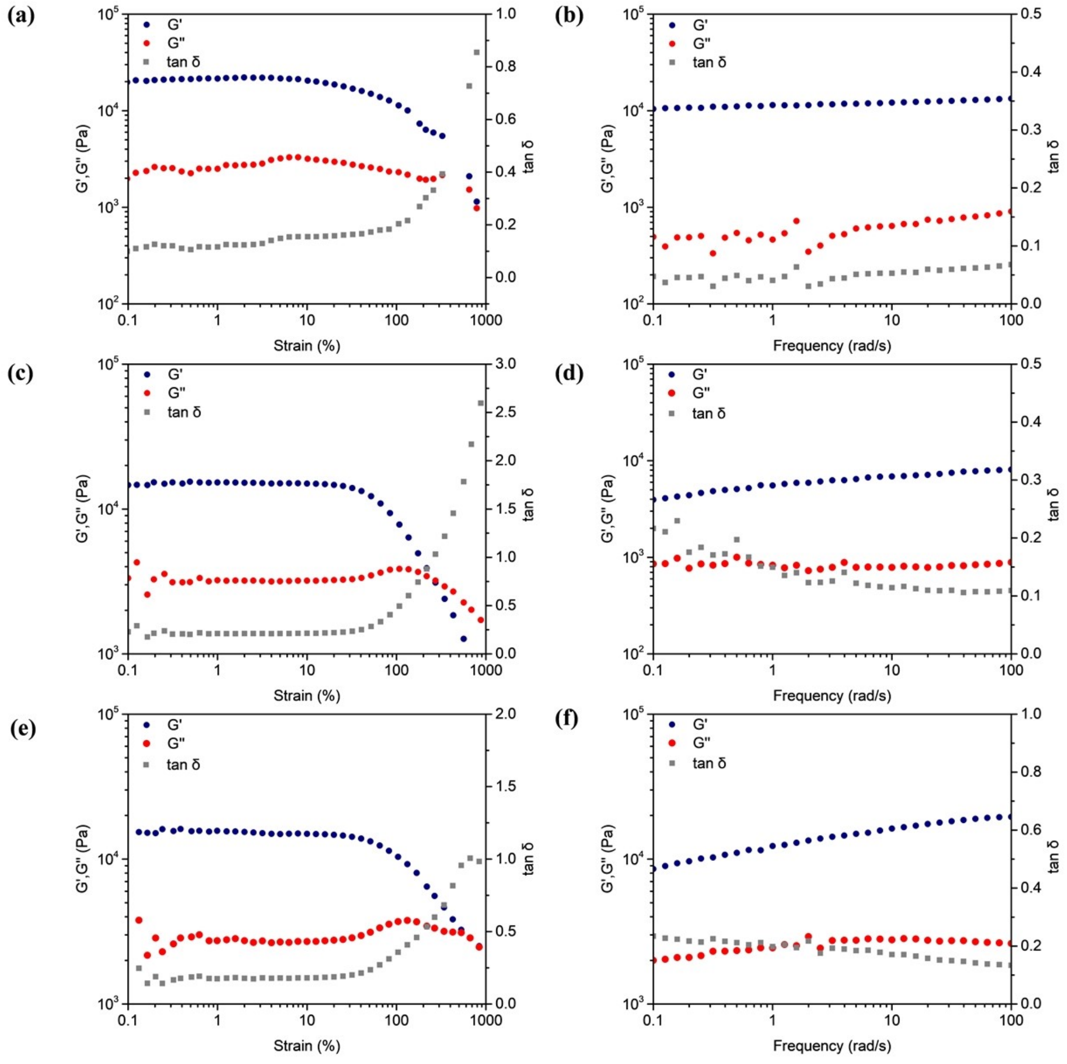

Figure S19. a) G′ (storage modulus) , G″(loss modulus) and tan δ (loss factor) as functions of strain (PEGDA/PEDOT:PSS/AM/AA hydrogel, with 0.4 wt% PEGDA). b) G′, G″ and tan δ as functions of frequency(PEGDA/PEDOT:PSS/AM/AA hydrogel, with 0.4 wt% PEGDA). c) G′, G″ and tan δ as functions of strain (PR-PEGMA$_3$/PEDOT:PSS/AM/AA hydrogel, with 0.4 wt% PR-PEGMA$_3$). d) G′, G″ and tan δ as functions of frequency (PR-PEGMA$_3$/PEDOT:PSS/AM/AA hydrogel, with 0.4 wt% PR-PEGMA$_3$). e) G′, G″ and tan δ as functions of strain (PR-PEGMA$_{12}$/PEDOT:PSS/AM/AA hydrogel, with 0.4 wt% PR-PEGMA$_{12}$). f) G′, G″ and tan δ as functions of frequency (PR-PEGMA$_{12}$/PEDOT:PSS/AM/AA hydrogel, with 0.4 wt% PR-PEGMA$_{12}$).

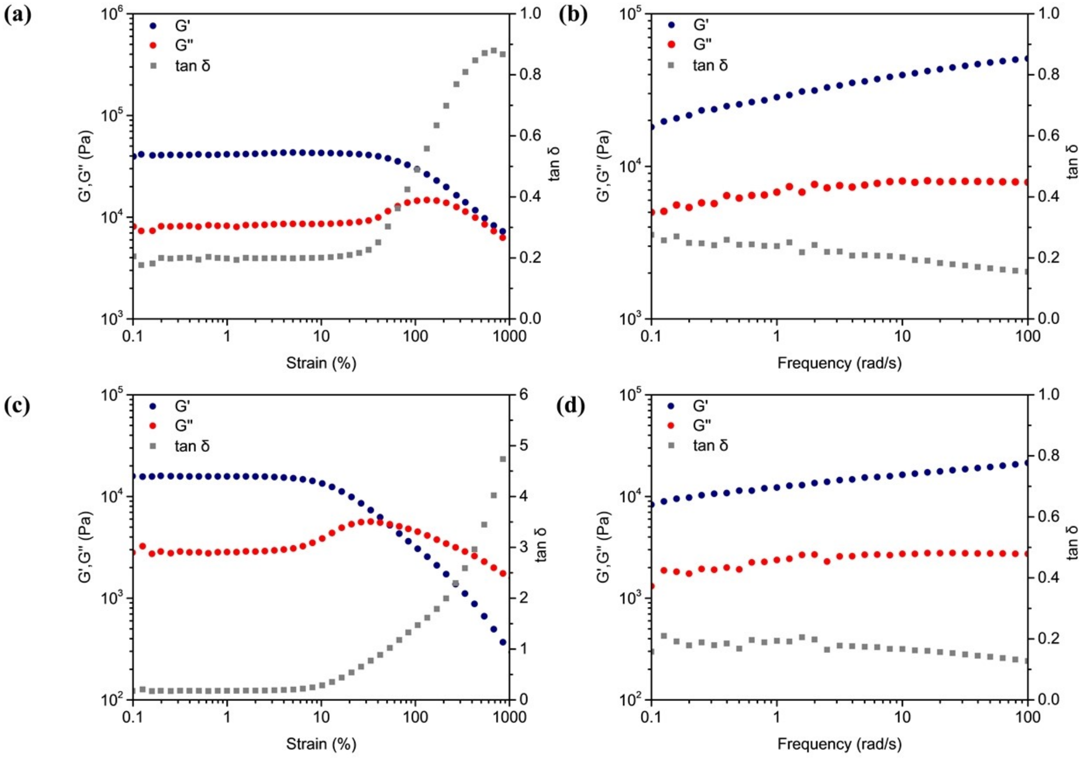

Figure S20. a) G′ (storage modulus) , G″(loss modulus) and tan δ (loss factor) as functions of strain (PR-PEGMA$_8$/PEDOT:PSS/AM/AA hydrogel, with 1.0 wt% PR-PEGMA$_8$). b) G′, G″ and tan δ as functions of frequency(PR-PEGMA$_8$/PEDOT:PSS/AM/AA hydrogel, with 1.0 wt% PR-PEGMA$_8$). c) G′, G″ and tan δ as functions of strain (PR-PEGMA$_8$/PEDOT:PSS/AM/AA hydrogel, with 1.6 wt% PR-PEGMA$_8$). d) G′, G″ and tan δ as functions of frequency (PR-PEGMA$_8$/PEDOT:PSS/AM/AA hydrogel, with 1.6 wt% PR-PEGMA$_8$).

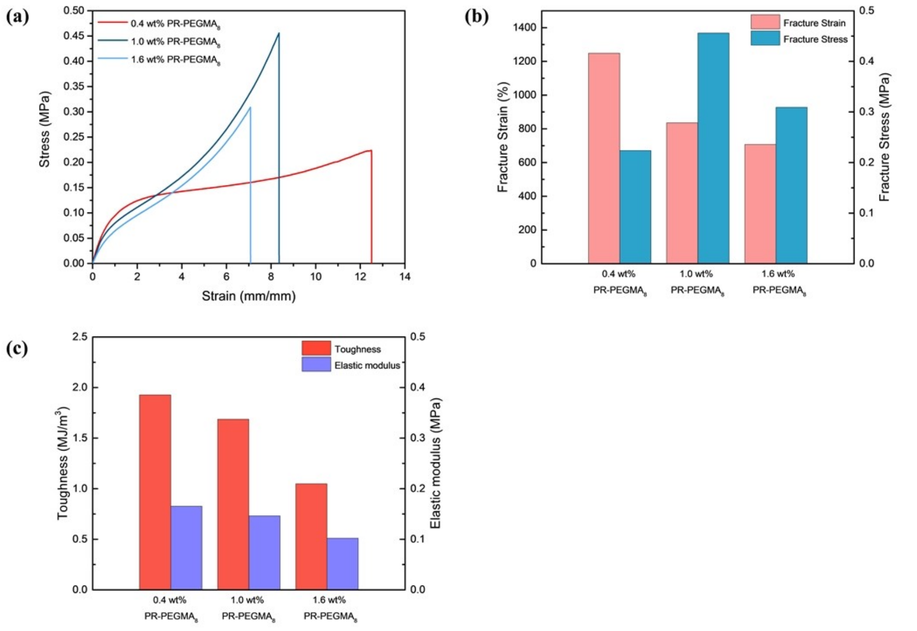

Figure S21. a) Tensile stress−strain curves of PR-PEGMA$_8$/PEDOT:PSS/AM/AA hydrogels with different cross-linker ratio (red, 0.4 wt%; dark blue, 1.0 wt%; light blue, 1.6 wt%). b) The fracture stresses and fracture strains of PR-PEGMA$_8$/PEDOT:PSS/AM/AA hydrogels with different cross-linker ratio. c) The tensile toughness and elastic moduli of PR-PEGMA$_8$/PEDOT:PSS/AM/AA hydrogels with different cross-linker ratio.

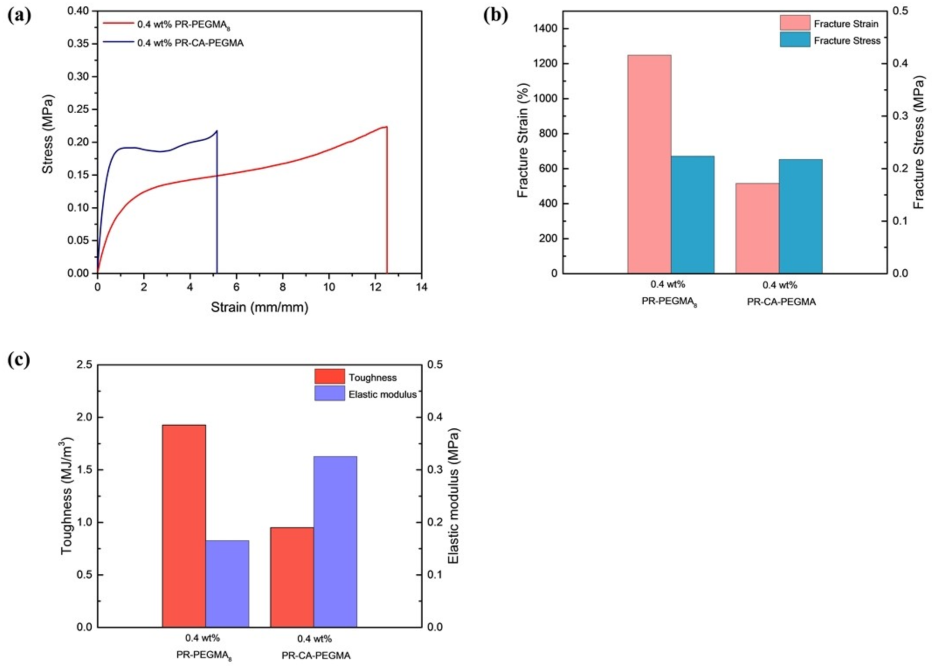

Figure S22. a) Tensile stress−strain curves of PR-PEGMA$_8$/PEDOT:PSS/AM/AA hydrogel (red) and PR-CA-PEGMA/PEDOT:PSS/AM/AA hydrogel (dark blue). b) The fracture stresses and fracture strains of PR-PEGMA$_8$/PEDOT:PSS/AM/AA hydrogel and PR-CA-PEGMA/PEDOT:PSS/AM/AA hydrogel. c) The tensile toughness and elastic moduli of PR-PEGMA$_8$/PEDOT:PSS/AM/AA hydrogel and PR-CA-PEGMA/PEDOT:PSS/AM/AA hydrogel.

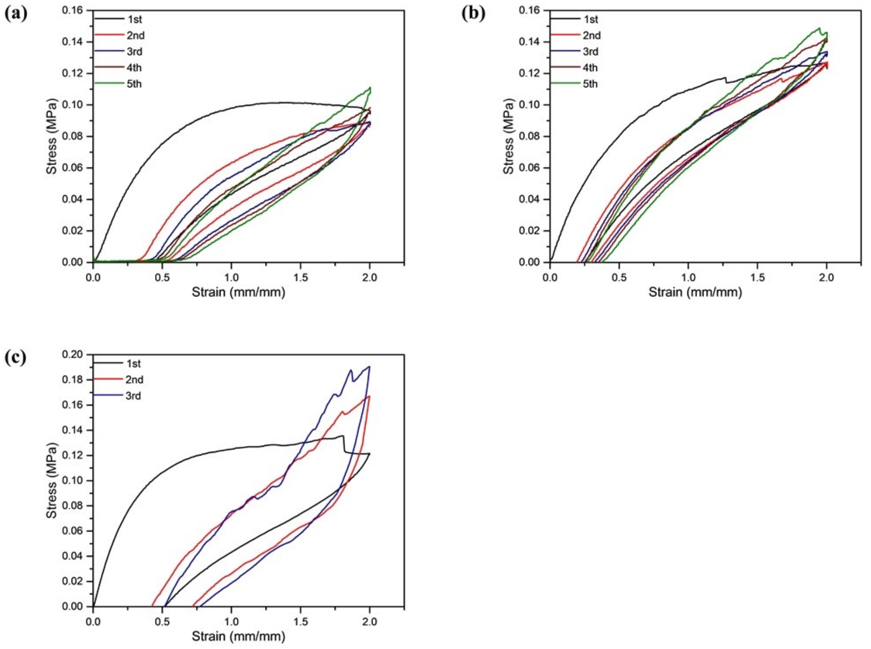

Figure S23. Tensile loading−unloading curves of a) PR-PEGMA$_3$/PEDOT:PSS/AM/AA hydrogel, b) PR-PEGMA$_{12}$/PEDOT:PSS/AM/AA hydrogel, and c) PR-CA-PEGMA/PEDOT:PSS/AM/AA hydrogel.

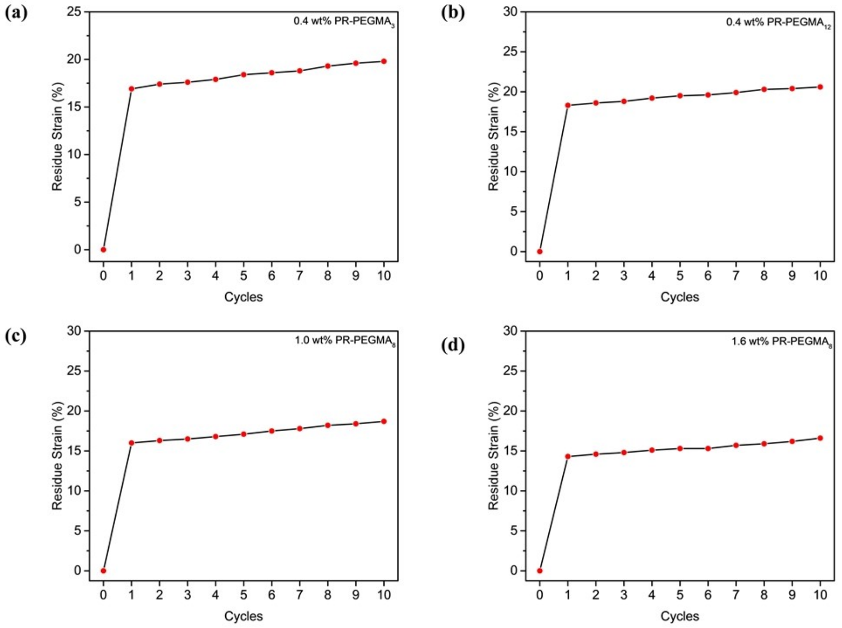

Figure S24. Residue strain of PR-PEGMA$_x$/PEDOT:PSS/AM/AA hydrogel as a function of cycle times at a strain of 200%, a) with 0.4 wt% PR-PEGMA$_3$, b) with 0.4 wt% PR-PEGMA$_{12}$, c) with 1.0 wt% PR-PEGMA$_8$, d) with 1.6 wt% PR-PEGMA$_8$.

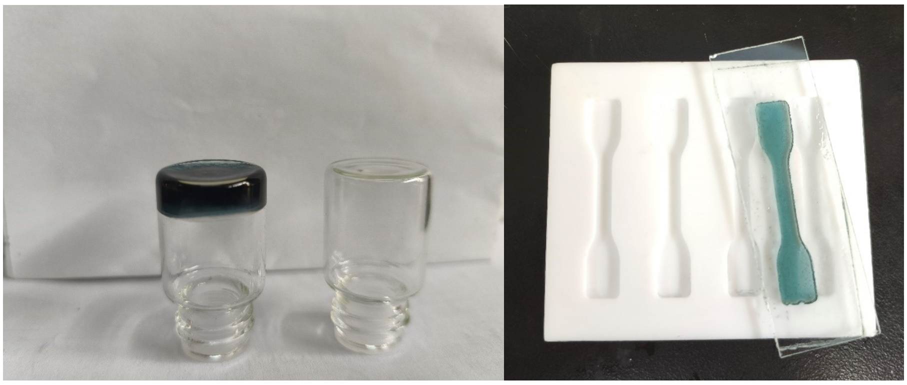

Figure S25. Image of the appearance of the hydrogel.

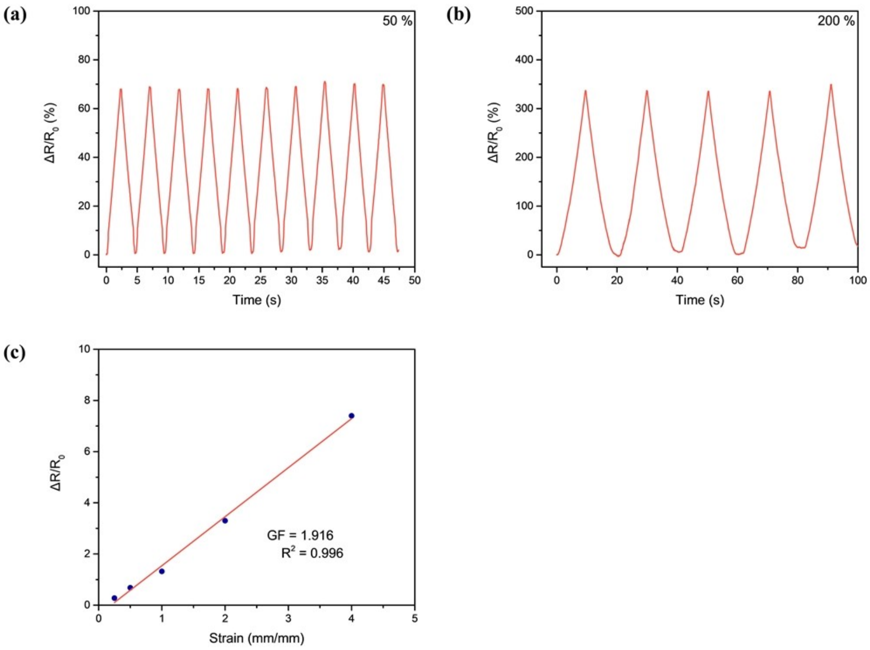

Figure S26. $\Delta R/R_0$ curves of the hydrogel during the stretching-recovery cycle when the tensile strain is a) 50 % and b) 200 %. c) The gauge factor (GF) of the hydrogel.

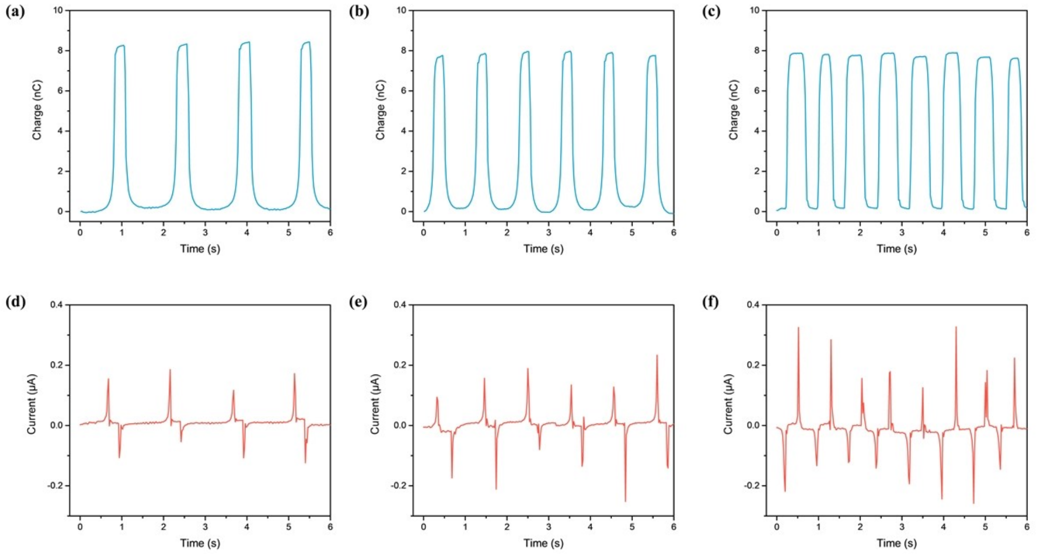

Figure S27. Q_{SC} of a PDMS-VHB single electrode TENG with a contact separation frequency of a) 0.67 Hz, b) 1.0 Hz and c) 1.4 Hz. I_{SC} of a PDMS-VHB single electrode TENG with a contact separation frequency of d) 0.67 Hz, e) 1.0 Hz and f) 1.4 Hz.

References

1 Y. Jiang, Z. Zhang, Y.-X. Wang, D. Li, C.-T. Coen, E. Hwaun, G. Chen, H.-C. Wu, D. Zhong, S. Niu, W. Wang, A. Saberi, J.-C. Lai, Y. Wu, Y. Wang, A. A. Trotsyuk, K. Y. Loh, C.-C. Shih, W. Xu, K. Liang, K. Zhang, Y. Bai, G. Gurusankar, W. Hu, W. Jia, Z. Cheng, R. H. Dauskardt, G. C. Gurtner, J. B.-H. Tok, K. Deisseroth, I. Soltesz and Z. Bao, Topological supramolecular network enabled high-conductivity, stretchable organic bioelectronics, *Science*, 2022, **375**, 1411-1417.

2 A. Bin Imran, K. Esaki, H. Gotoh, T. Seki, K. Ito, Y. Sakai and Y. Takeoka, Extremely stretchable thermosensitive hydrogels by introducing slide-ring polyrotaxane cross-linkers and ionic groups into the polymer network, *Nat. Commun.*, 2014, **5**, 5124.
